# Cyclopentadienyl–Silsesquioxane Titanium Complexes in the Polymerizations of Styrene and L-Lactide

**DOI:** 10.3390/polym17192715

**Published:** 2025-10-09

**Authors:** Joan Vinueza-Vaca, Shoaib Anwar, Salvatore Impemba, Ilaria Grimaldi, Gerardo Jiménez, Carmine Capacchione, Vanessa Tabernero, Stefano Milione

**Affiliations:** 1 Department of Organic and Inorganic Chemistry, Alcalà University, Campus Universitario, 28871 Alcala de Henares, Spain; 2 Department of Chemistry and Biology, University of Salerno, Via Giovanni Paolo II, 84084 Fisciano, Italy; sanwar@unisa.it (S.A.); simpemba@unisa.it (S.I.); ilagrimaldi@unisa.it (I.G.);

**Keywords:** ring-opening polymerization, Ziegler-Natta polymerization, silsesquioxane titanium complex, cyclopentenyl complex, density functional theory

## Abstract

In this contribution, two silsesquioxane–cyclopentadienyl titanium complexes featuring one or two chloride ancillary ligands, [Ti(η^5^-C_5_H_4_SiMeO_2_Ph_7_Si_7_O_10_-*κ*O)Cl_2_] (**1**) and [Ti(η^5^-C_5_H_4_SiMe_2_OPh_7_Si_7_O_11_-*κ*^2^O_2_)Cl] (**2**), were synthesized and evaluated in the Ziegler–Natta polymerization of styrene and the ring-opening polymerization (ROP) of L-lactide, respectively. Complex **1**, activated with methylaluminoxane (MAO), catalyzed the syndiotactic polymerization of styrene with turnover frequencies up to 28 h^−1^, affording polymers with narrow dispersity, low number-average molecular weights (*M*_n_ = 5.2–8.2 kDa), and high stereoregularity, as confirmed by ^13^C NMR. Complex **2**, in combination with benzyl alcohol, promoted the ring-opening polymerization of L-lactide in solution at 100 °C, achieving conversions up to 95% with good molecular weight control (*M*_n_ close to theoretical, *Đ* = 1.19–1.32). Under melt conditions at 175 °C, it converted up to 3000 equiv. of monomer within 1 h. Kinetic analysis revealed first-order dependence on monomer concentration. The results highlight the ability of these complexes to produce syndiotactic polystyrene with narrow molecular weight distributions and to catalyze controlled ROP of L-lactide under both solution and melt conditions. Computational studies provided insight into key structural and energetic features influencing reactivity, offering a framework for further catalyst optimization. This work broadens the application scope of silsesquioxane–cyclopentadienyl titanium complexes and supports their potential as sustainable and versatile catalysts for both commodity and biodegradable polymer synthesis.

## 1. Introduction

The Ziegler–Natta (ZN) and ring-opening polymerization (ROP) processes have played pivotal roles in advancing the field of material science. The ZN polymerization has enabled the synthesis of polymers with monomeric distributions and stereosequences that were previously unattainable [1,2,3,4]. Thanks to its efficiency in polymerizing low-cost monomers, ZN polymerization has become of fundamental importance for large-scale production of commodity plastics [5,6]. In recent years, growing environmental concerns related to the use of petroleum-based feedstocks and the accumulation of plastic waste have prompted the development of ROP of cyclic esters as this polymerization technique utilizes renewable monomers and enables the production of biodegradable polymers [7,8]. Although ZN and ROP are mechanistically distinct, both are efficiently and selectively catalyzed by transition metal complexes. In both cases, the catalyst activates the monomer through coordination to a Lewis acidic metal centre, thereby facilitating its subsequent insertion into the growing polymer chain.

Group 4 cyclopentadienyl and metallocene complexes have been extensively employed in olefin polymerization [1,9,10,11,12] and a range of other catalytic processes [13]. However, their application in lactide polymerization remains relatively limited [14,15,16,17]. The first report came from Chen and co-workers, who found that zirconocenes with *C*_2_- and *C*_2v_-symmetry displayed only marginal activity, while the bis(ester enolate) zirconocene Ph_2_C(Cp)(Flu)Zr[OC(O^i^Pr)=CMe_2_]_2_ afforded isotactic PLA with narrow dispersity [18]. Pitsikalis and co-workers employed the half-sandwich titanium complex CpTiCl_2_(OEt), which proved to be a slow initiator (achieving full conversion after 48 h at 130 °C) unless combined with the Lewis acidic CpTiCl_3_ [19,20,21].

As part of our ongoing research, we have been interested in the silsesquioxane–cyclopentadienyl titanium complexes. Simple silsesquioxane titanium complexes have attracted attention as they can be considered molecular models for siliceous heterogeneous catalysts [22,23,24,25,26,27], and several have been explored for olefin polymerization [28,29,30,31,32,33,34]. Linked silsesquioxane–cyclopentadienyl titanium complexes can be considered as derivatives of half-titanocenes. Previous studies have demonstrated that fine-tuning the electronic and steric properties of the cyclopentadienyl ligand, as well as modifying the monodentate anionic donor ligand, can significantly influence catalytic activity, polymer molecular weights, and stereoregularity in the Ziegler–Natta polymerization. Notably, Nomura et al. showed that half-titanocenes bearing aryloxo ligands display higher catalytic activity in syndiospecific styrene polymerization than their trichloro analogues [35,36,37].

**Scheme 1:**
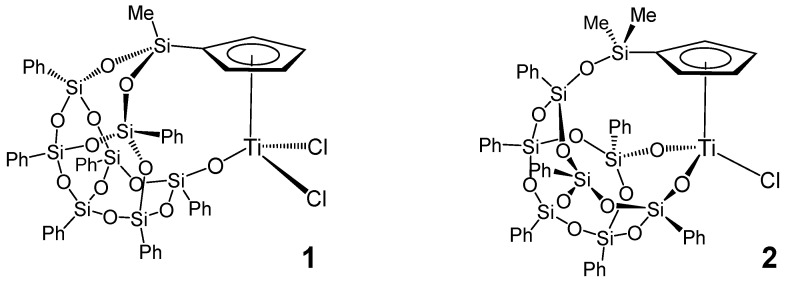
Structures of the silsesquioxane–cyclopentadienyl titanium complexes used as catalysts in this study.

Due to the non-toxicity and relative abundance in the Earth’s crust, titanium complexes are particularly appealing for the development of new sustainable catalysts [38]. For these reasons, in this study, we investigated the catalytic performance of two silsesquioxane–cyclopentadienyl titanium complexes (**1** and **2** in Scheme 1) in the Ziegler–Natta polymerization of styrene and the ring-opening polymerization (ROP) of L-lactide. Complex **1**, bearing two chloride ancillary ligands, was synthesized for the first time in this work and thoroughly characterized by NMR spectroscopy. In contrast, complex **2** had been previously prepared and evaluated in the epoxidation of olefins as well as in the polymerization of myrcene and anethole [39,40,41]. Complex **1** demonstrated moderate activity toward the formation of highly syndiotactic polystyrene, while complex **2** efficiently promoted controlled ROP of L-lactide in the presence of benzyl alcohol. Density functional theory (DFT) studies were employed to elucidate the polymerization mechanisms, providing mechanistic insights into the nature of the active species. To the best of our knowledge, this is the first application of silsesquioxane–cyclopentadienyl titanium complexes in lactide ROP. Altogether, this study expands their application scope and underscores their potential as sustainable and versatile catalysts for the synthesis of both commodity and biodegradable polymers.

## 2. Results and Discussion

### 2.1. Synthesis of the Complex [Ti(η^5^-C_5_H_4_SiMeO_2_Ph_7_Si_7_O_10_-κO)Cl_2_] (**1**)

**Scheme 2:**
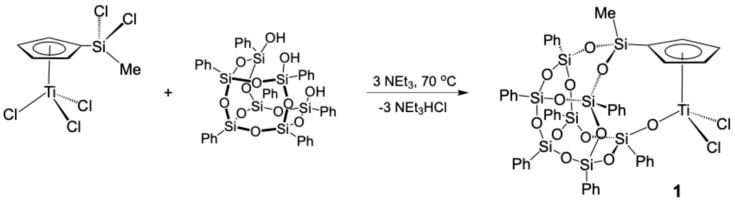
Synthesis of [Ti(η^5^-C_5_H_4_SiMeO_2_Ph_7_Si_7_O_10_-*κ*O)Cl_2_] (**1**).

The complex [Ti(η^5^-C_5_H_4_SiMeO_2_Ph_7_Si_7_O_10_-*κ*O)Cl_2_] (**1**) was prepared following a synthetic strategy previously reported by our research group for the synthesis of the silsesquioxane–cyclopentadienyl titanium complex [Ti(η^5^-C_5_H_4_SiMe_2_OPh_7_Si_7_O_11_-*κ*^2^O_2_)Cl] (**2**) (Scheme 1) [42]. In order to enable a double assembly between the Cp ring and the silsesquioxano moiety and, in this way, to obtain the complex **1** with a structure related to the conventional Ziegler–Natta catalyst, we proposed the monocyclopentadienyltitanium derivative [Ti(η^5^-C_5_H_4_SiMeCl_2_)Cl_3_] as the starting material. It was envisaged that the presence of a second chloride atom bonded to the silicon atom would allow for a second isomerization process, similar to the one observed in the formation of compound **2**. In this context, an equimolar reaction between [Ti(η^5^-C_5_H_4_SiMeCl_2_)Cl_3_] and the condensed silsesquioxane trisilanol Ph_7_Si_7_O_9_(OH)_3_, in the presence of NEt_3_, in chloride solvents, and at 70 °C, furnished the doubly connected silsesquioxane–cyclopentadienyl compound [Ti(η^5^-C_5_H_4_SiMeO_2_Ph_7_Si_7_O_10_-*κ*O)Cl_2_] (**1**) (Scheme 2).

Complex **1** was successfully isolated as a yellow solid in high yield and was fully characterized spectroscopically and analytically. The spectroscopic behaviour of **1** indicates a *C*_s_ symmetry as the ^1^H-NMR spectrum shows an AA’BB’ spin system for each pair of equivalent proximal and remote Cp protons, along with the corresponding resonances for the phenylic protons of the silsesquioxano moiety. Further, the silicon chemical shift for Cp-*Si* is key for establishing the structure of this complex, since it relates to the nature of the substituents of the silicon atoms. In this case, the silicon resonance appears at high field (−34 ppm) confirming the substitution of both chloride atoms which are initially bonded to the silicon, and therefore, the formation of the doubly connected silsesquioxane–cyclopentadienyl compound **1**.

**Scheme 3:**
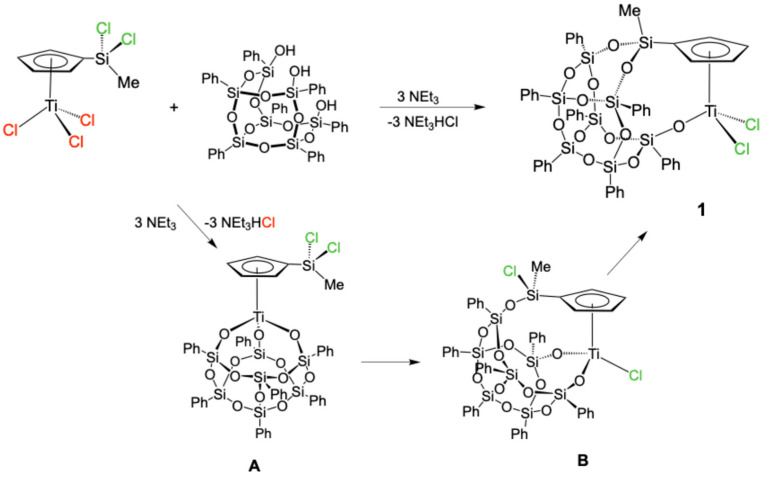
Synthetic route to the proposed intermediate titanosilsesquioxane species **A**, **B**, and target compound **1**.

To study the course of the reaction, the same reaction was performed at NMR-tube level, using a nonpolar solvent and at room temperature. In this experiment the formation of a mixture of three complexes, one of which is the complex **1**, was immediately observed. When the reaction mixture was kept at room temperature for a prolonged period of time, the resonances due to the two new compounds started to slowly disappear, finally furnishing a single set of resonances of the corresponding target compound, [Ti(η^5^-C_5_H_4_SiMeO_2_Ph_7_Si_7_O_10_-*κ*O)Cl_2_] (**1**). The two observed intermediate compounds are proposed to be [Ti(η^5^-C_5_H_4_SiMeCl_2_)(Ph_7_Si_7_O_12_-*κ* ^3^O_3_)] (**A**) and [Ti(η^5^-C_5_H_4_SiMeClOPh_7_Si_7_O_11_-*κ* ^2^O_2_)Cl] (**B**) formed during the reaction via two consecutive isomerization processes (Scheme 3).

Attempts to prepare and isolate the proposed intermediate compounds **A** and **B** were unsuccessful; however, their spectroscopic data are sufficiently clear to formally propose their structures. The NMR data for **A** indicates a *C*_s_ arrangement as the ^1^H-NMR spectrum shows an AA’BB’ spin system for the Cp ring protons, while those for the other intermediate compound, **B**, suggest a *C*_1_ symmetry, based on the observed ABCD spin system for the Cp ring protons. These spectroscopic features are in agreement with the proposed structures for **A**, a cubane-type species, and for **B**, a silsesquioxane–cyclopentadienyl compound arising from the replacement of only one of these, which results in the generation of a stereogenic centre on the silicon atom.

**Figure 1:**
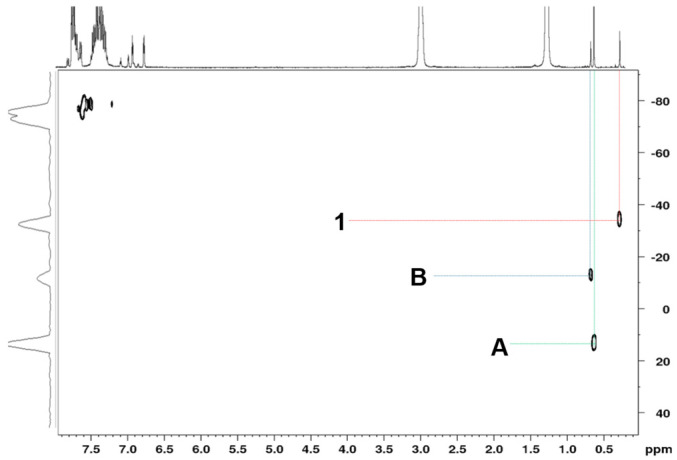
^1^H-^29^Si HMBC NMR spectrum of the crude reaction mixture displaying the presence of the two proposed intermediate compounds **A** and **B** and target compound **1**.

Once again, the silicon chemical shifts are also informative for establishing the structure of this type of compound, since this shift is affected by the number of chlorine atoms bonded to the silicon atom. It is observed that the silicon resonance is shifted towards higher field as the number of chlorine atoms bonded to the silicon atom decreases, as shown in Figure 1, appearing at 13, −13, and −34 ppm for compounds **A**, **B**, and **1**, respectively.

### 2.2. Styrene Polymerization

Complex **1** was tested in styrene polymerization after activation with methylaluminoxane (MAO) in toluene solution. The main results are summarized in Table 1.

The experiments were initially conducted at 60 °C using a fixed cocatalyst/catalyst molar ratio of 500, while varying the reaction time. After 3 h, the monomer conversion was relatively low but increased with extended polymerization time, reaching satisfactory conversion after 18 h. Raising the reaction temperature from 60 °C to 80 °C further enhanced the polymer yield. Although direct comparisons of catalytic activity are difficult due to differing experimental conditions, complex **1** displayed lower activity than CpTiCl_3_ [43] and the arloxo-substituted half-titanocenes reported by Nomura [35] but higher activity than the half-titanocenes with linked ethoxy–cyclopentadienyl ligand reported by Longo [43].

**Figure 2:**
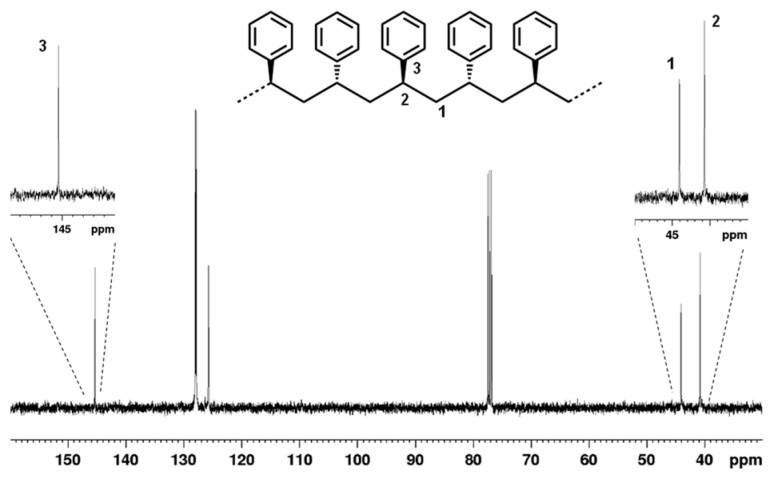
^13^C NMR spectrum of the polystyrene sample obtained in entry 4, Table 1. (CDCl_3_, rt, 150 MHz).

The resulting polystyrenes were soluble in chloroform. GPC analysis monomodal molecular weight distributions had dispersity values (Đ) close to 2, suggesting that a single dominant active species is responsible for polymerization. The number-average molecular weights (*M*_n_) were low and ranged from 6.1 to 8.2 *k*Da likely due to a slow propagation rate. Changing the cocatalyst/catalyst molar ratio did not significantly influence the chain length. To determinate the polymer microstructure, the obtained polymers were analyzed by ^13^C NMR spectroscopy. Figure 2 shows the ^13^C NMR spectrum of the polymer in entry 1. The signals corresponding to the phenyl *ipso*carbons and methylene carbons were observed at 145.35 and 44.04 ppm, respectively, and indicated a syndiotactic arrangement of the monomeric units [44]. The observed resonance pattern supports the formation of a highly regio- and stereoregular polymer structure.

It has been reported that half-titanocene catalysts, when activated with methylalumoxane, are reduced to species containing the titanium atom in various oxidation states such as Ti(III) and Ti(II) [45,46]. Supported by ESR studies, Grassi et al. proposed that the active species for the syndiotactic styrene polymerization is the *cationic* Ti(III) species with general formula Cp*TiR^+^ [47,48,49,50]. This hypothesis was experimentally supported by Waymouth and Mahanthappa who investigated the styrene polymerization catalyzed by Cp*Ti^III^(C_3_H_5_)_2_-[PhN(H)Me_2_][B(C_6_F_5_] or Cp*Ti^III^(C_3_H_5_)_2_-[Ph_3_C][B(C_6_F_5_)_4_] and demonstrated that Ti(IV) species are not catalytically active [51]. In contrast, the Ti(IV) species present during the polymerization has been proposed as the plausible active species either for ethylene polymerization or styrene–ethylene copolymerization [52]. Regarding the half-titanocenes containing an aryloxo ligand, Nomura et al. suggested that the *neutral* Ti(III) species of the Cp*Ti(OAr)R type is responsible for syndiotactic styrene polymerization, a conclusion supported by in situ solution X-ray absorption fine structure (XAFS) studies. It was observed that upon treating Cp’TiCl_2_(OAr) (Cp’ = Cp or ^t^BuC_5_H_4_) with MAO in the presence of styrene, the Ti(IV) complex was reduced and the Ti-O bond was preserved along with dissociation of Ti-Cl bonds [53,54]. DFT calculation gave further support to the hypothesis that the active species is a neutral half-metallocene Ti(III) species [55].

**Figure 3:**
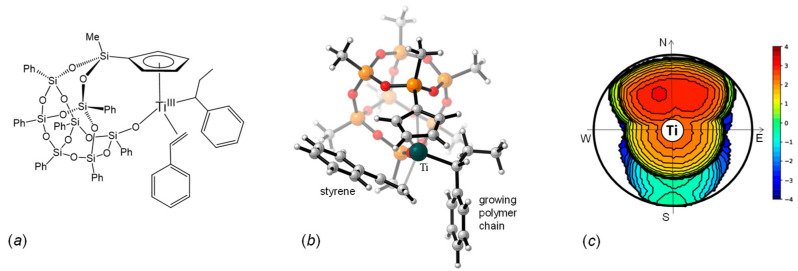
The proposed active species for the syndiotactic styrene polymerization (**a**), the DFT optimized structure (**b**), and the steric encumber determined by the cyclopentadienyl–silsesquioxane ligand (**c**). The isocontour curves are given in Å. (%V_bur_ = 69.5).

Based on these results we assumed that a possible active species in our system is the natural Ti(III) complex shown in Figure 3a. In this structure, the titanium atom is bound to the cyclopentadienyl, to the anionic oxygen atom of the silsesquioxane, to the growing polymeric chain, and to the styrene molecule. The DFT optimized structure is depicted in Figure 3b. The steric environment imposed by the cyclopentadienyl–silsesquioxane ligand at the active site is shown in Figure 3c [56]. The cyclopentadienyl ligand lies horizontally across the upper hemisphere, while the two potential coordination sites are oriented toward the southeast and southwest quadrants of the sphere. The active species exhibits the expected *C*_s_-symmetric structure, prone to syndiotactic enchainment. The two diasterotopic coordination sites are relatively accessible, with calculated buried volumes of 43.2% and 44.9% for the southwest and southeast quadrants, respectively.

In our calculations, we explored the coordination of styrene at both active sites, orienting the monomer and the growing polymer chain to allow for secondary monomer insertion into the Ti–alkyl bond. Transition state structures were located for the syndiotactic and isotactic insertion pathways but their energies were nearly degenerate, making it difficult to rationalize the experimentally observed stereoselectivity. This limitation likely reflects the approximations inherent in the employed DFT model; however, a more detailed investigation of these factors lies beyond the scope of the present study.

### 2.3. Lactide Polymerization

Complex **2** was evaluated as a catalyst for the ring-opening polymerization (ROP) of *L*-lactide (*L*-LA) under various experimental conditions. Representative results are summarized in Table 2. At 100 °C, in toluene solution, complex **2** exhibited negligible activity likely due to the limited initiating ability of its chloride ligand. The addition of benzyl alcohol as a cocatalyst, enhanced its catalytic performance. In particular, high monomer conversions were reached in 20 h using one or two equivalents of benzyl alcohol, or in 16 h using three equivalents of benzyl alcohol. Lowering the polymerization temperature resulted in a marked decrease in activity (see Figure S7 in Supporting Information).

**Figure 4:**
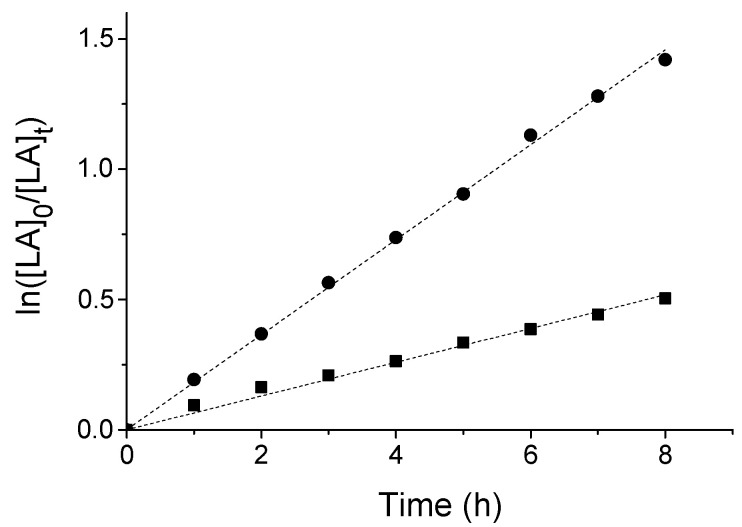
Pseudo-first-order kinetic plots for ROP of *L*-LA in presence **2** and one (*k*_app_ = 0.064 ± 0.001 h^−1^, *R*^2^ = 0.996, ●) or three equivalents of BzOH (*k*_app_ = 0.182 ± 0.001 h^−1^, *R*^2^ = 0.999, ■). Conditions: [LA]_0_ = 1.0 M; [LA]_0_/[**2**]_0_ = 150, T = 100 °C, toluene (2.0 mL) as solvent.

To gain further insight into the polymerization kinetic, the reaction promoted by **2**/BzOH at 100 °C was monitored at regular time intervals and the corresponding kinetic plot was constructed. As illustrated in Figure 4 the polymerization starts immediatly and proceeds with a first-order kinetic with respect to the monomer concentration. The apparent propagation rate constant (*k*_app_) was determined to be 0.064 ± 0.001 h^−1^ for one equivalent of BzOH and 0.182 ± 0.001 h^−1^ for three equivalents.

The resulting polymers exhibited monomodal molecular weight distributions. The number-average molecular weights were close to those expected, based on the assumption of one polymer chain growing per equivalent of benzyl alcohol. The dispersity was in the range 1.19–1.32 suggesting that the polymerization is influenced by intra- and intermolecular chain-transfer side reactions. Investigation of the relationship between the number-average molecular weights and monomer conversion revealed that the *M*_n(exp)_ increased linearly with conversion, in excellent agreement with expected values (Figure S8).

The good match between the *M*_n(exp)_ and the theoretical values, together with the monomodal and narrow dispersity and the linear increase in the *M*_n(exp)_ suggest that the polymerization catalyzed by **2**/BzOH allows the production of polymers with predictable sizes and reproducible structures.

To investigate the polymerization mechanism, a polylactide sample with low molecular weight was prepared and analyzed. The ^1^H NMR spectrum (Figure S10, Supporting Information) displayed a quartet at 4.33 ppm, corresponding to the methine protons of the lactyl end group. The presence of the benzyloxy end group was revealed by the inspection of the ^13^C NMR spectrum, which showed a signal at 67.4 ppm corresponding to the methine carbon atom of the benzyloxy group, along with four signals in the range 120–140 ppm. These assignments were confirmed by MALDI-TOF mass spectrometry, which revealed polymer chains terminated with the same end groups. Additionally, the detected mass increments of 72 u suggest that both inter- and intramolecular transesterification side reactions affect the propagation process.

End-group analysis indicates that polymerization is initiated by a nucleophilic attack of the benzyloxy group on the carbonyl carbon of the monomer, following a coordination–insertion mechanism. Chain termination occurs via hydrolytic cleavage of the Al–O bond connecting the polymer chain to the metal centre.

The catalytic performances were investigated under more industrially relevant conditions: in melt monomer at 175 °C using technical grade monomers. In conditions, the complex was able to convert 1500 or 3000 equivalents of L-LA in one hour. The *M*_n_ values were lower than the theoretical values, and the dispersity was relatively broad, suggesting a loss of control under these experimental conditions.

**Figure 5:**
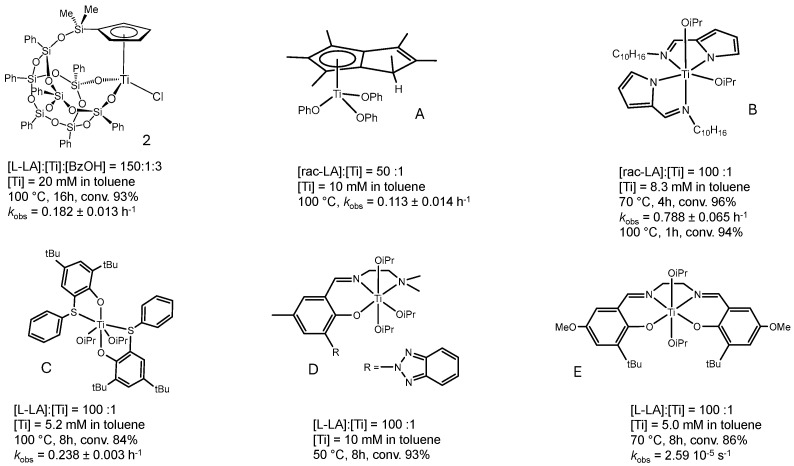
Catalytic activity of selected group IV complexes in the ROP of cyclic esters.

Although the activity of complex **2** is lower than that of some recently reported zirconium complexes [57,58], it is comparable to the values typically observed for titanium-based catalysts [14,15,16,17]. Figure 5 provides a comparative overview of the catalytic performance of selected titanium complexes; this comparison is restricted to catalytic activities evaluated under solution-phase conditions. Complex A [59], a cyclopentadienyl derivative, was included due to its structural similarity to **2**. Complex B [60] was selected as it is, to the best of our knowledge, among the most active titanium-based catalysts reported, to date: at 70 °C, it allowed 96% monomer conversion to be reached in 4 h. Complexes C [61], D [62], and E [63] were chosen as representative examples of titanium complexes bearing bi-, tri-, and tetradentate ligands. The activity of these complexes is roughly double that of complex **2**. Therefore, complex **2** displays higher catalytic activity than its structural analogue, complex A. However, both complexes generally exhibit lower activity compared to titanium systems featuring non-cyclopentadienyl ligands.

**Figure 6:**
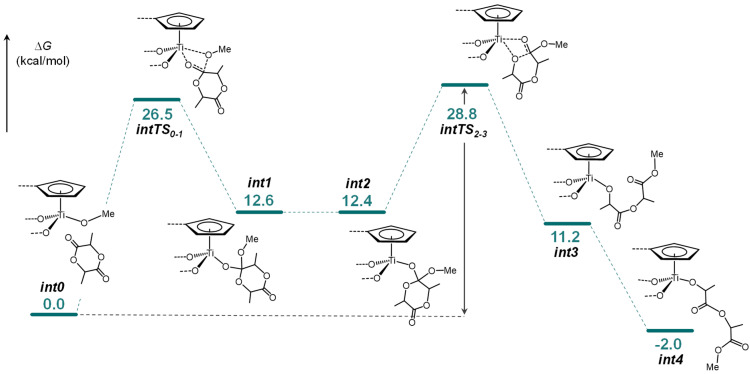
DFT free energy profile for the ring-opening polymerization of L-LA promoted by **2**/BzOH (BP86-D3/TZVP/Lanl2DZ-CPCM(toluene)//BP86/6-31G(d)/Lanl2DZ).

**Figure 7:**
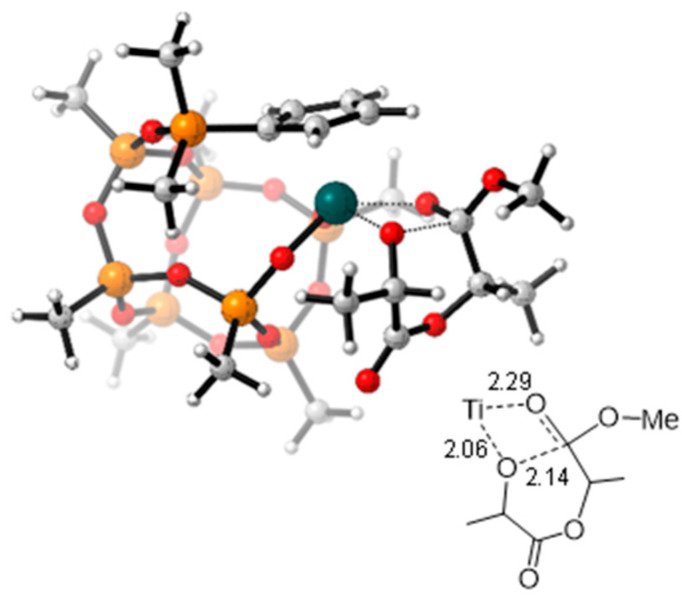
Minimum energy structure for the transition states ***intTS_2-3_***. The distances are given in Angstrom.

The free energy reaction pathway for the ring-opening polymerization of L-LA was investigated computationally. All attempts to localize a starting intermediate featuring the direct coordination of L-LA to the Ti atom were unsuccessful. Instead, a starting adduct (***int0***) was identified, in which the monomer is docked onto the ligand through noncovalent interactions. On the free energy surface, the formation of this structure is lightly disfavoured, most likely due to the entropy decrease associated with its formation. The binding enthalpy (Δ*H_bind_*) for ***int0***, calculated as the difference between the enthalpy of the adduct and the sum of the enthalpies of **2** and the monomer, was −11.8 kcal/mol. Taking ***int0*** as the starting point, the reaction proceeds via a two-step mechanism (Figure 6). In the first step, the system evolves through a Transition State ***TS_0-1_*** that is located at 27.2 kcal/mol on the reaction profile. Beyond ***TS_0–1_***, the resulting orthoalkoxide intermediate ***int1*** undergoes rearrangement, bringing the intracyclic oxygen atom of the LA closer to the metal centre. From ***int2***, the ring opening occurs through transition state ***TS_2–3_*** (Figure 7), located at 29.5 kcal/mol, to afford the intermediate ***int3***. Finally, the isomerization of the growing chain yields to the stable heterocyclic Ti lactate product (***int4***) rendering the ring-opening reaction of the LA thermodynamically favoured. The ring-opening step via ***TS_2–3_*** represents the rate-determining step of the overall process (Figure 6). The relatively high activation barrier of 28.8 kcal/mol is consistent with the requirement for elevated polymerization temperatures and explains the catalytic activity observed for complex **2**.

## 3. Conclusions

In this study two silsesquioxane–cyclopentadienyl titanium complexes were evaluated in the polymerization of styrene and L-lactide. Complex **1** with two Ti-Cl bonds, when activated with methylaluminoxane (MAO), exhibited moderate activity in syndiotactic styrene polymerization. The obtained polystyrenes exhibited narrow dispersity and high stereoregularity, indicating that a single, well-defined active species is likely involved. Although DFT calculations suggest a plausible neutral Ti(III) species as the catalyst, the observed stereoselectivity could not be fully rationalized, indicating that the active species may undergo further structural transformations under polymerization conditions.

Complex **2**, with one Ti-Cl bond, was active in the ring-opening polymerization of L-lactide in the presence of benzyl alcohol as a cocatalyst. The reaction followed a first-order kinetic profile with respect to monomer concentration and exhibited good control over molecular weights and dispersity. Mechanistic studies and DFT calculations support a coordination–insertion mechanism initiated by the nucleophilic attack of the benzyloxy group, with the ring-opening transition state representing the rate-determining step. Although the activation barrier is relatively high, the catalytic performance of complex **2** is comparable to other titanium-based systems.

Overall, the results underscore the potential of silsesquioxane–cyclopentadienyl titanium complexes as versatile catalysts for both olefin and cyclic ester polymerizations.

## 4. Experimental Section

The description of materials, method, instruments, and measurements is provided in the Supporting Information. Complex [Ti(η^5^-C_5_H_4_SiMe_2_OPh_7_Si_7_O_11_-*κ*^2^O_2_)Cl] (**2**) was synthesized according to the literature procedures [42].

### 4.1. Synthesis of [Ti{η^5^-C_5_H_4_SiMeO_2_Ph_7_Si_7_O_10_-κO}Cl_2_] (**1**)

An amount of 0.3 g of compound [Ti(η^5^-C_5_H_4_SiMeCl_2_)Cl_3_] (10.1016/S0022-328X(97)00518-4) (0.90 mmol) and 0.84 g of Ph_7_Si_7_O_9_(OH)_3_ (0.90 mmol) were weighed into an ampoule, dissolved in dichloromethane, and 3 equivalents of triethylamine (NEt_3_) (0.4 mL, 2.70 mmol) were added. The mixture was then heated at 70 °C overnight under constant stirring. The solvent was subsequently evaporated, and the resulting residue was extracted with toluene, with the ammonium salt being removed by filtration. The resulting solution was dried, yielding a yellow solid, which was washed with hexane to obtain **3** with an 83% yield (0.8 g, 0.75 mmol). ^1^H-NMR (C_6_D_6_): d 0.24 (s, 3H, Si**Me**O_2_), 6.36, 7.01 (m, AA`BB, 4H, C_5_**H_4_**SiMeO_2_), 7.01–7.18, 7.80–8.02 (C_6_H_5_); ^13^C{^1^H} NMR (C_6_D_6_): d 0.32 (Si**Me**O_2_), 124.3, 127.8 (**C**_5_H_4_SiMeO_2_), 125.7 C*_ipso_* (**C**_5_H_4_SiMeO_2_), 128.4–131.5, 134.3–134.9 (**C**_6_H_5_); HMBC ^1^H-^29^Si (C_6_D_6_): d −34 ppm (Si**Me**O_2_), −76.0, −77.6, −79.3 (Ph_7_**Si_7_**O_12_). Anal. found: C, 50.1; H, 3.76. Calc for C_48_H_42_Cl_2_O_12_Si_8_Ti: C, 49.95; H, 3.67.

### 4.2. Typical Procedure for Styrene Polymerization

In a glovebox, a Schlenk flask (50 cm^3^) was sequentially charged with complex [Ti(η^5^-C_5_H_4_SiMeO_2_Ph_7_Si_7_O_10_-*κ*O)Cl_2_] (**1**) (12 mg, 10^−5^ mol), which was then dissolved in toluene (8.0 mL), followed by the addition of a 10% MAO solution in toluene (2.2 mL). The resulting mixture was stirred for 15 min at room temperature. Subsequently, styrene (0.6 mL) was added, and the reaction mixture was immersed in a thermostated oil bath at the desired temperature for a defined period. Upon completion, the polymerization mixture was poured into acidified ethanol (100 mL) to quench the reaction. The resulting polymer was collected by filtration and dried under vacuum at 60 °C overnight.

### 4.3. Typical Procedure for L-Lactide Polymerization

In a glovebox, a Schlenk flask (10 cm^3^) was charged sequentially with *L*-lactide, complex [Ti(η^5^-C_5_H_4_SiMe_2_OPh_7_Si_7_O_11_-*κ*^2^O_2_)Cl] (**2**), and solvent (2.0 mL). The mixture was heated thermostatically at the required temperature. At specified time intervals, a small amount of the polymerization mixture was sampled by using a pipette and quenched in wet CDCl_3_. This fraction was subjected to monomer conversion determination, which was monitored by the integration of monomer versus polymer methine resonances in the ^1^H NMR spectrum (CDCl_3_) at 25 °C. After the required polymerization time, the reaction mixture was quenched with wet *n*-hexane. The obtained polymer was collected by filtration and dried in vacuum at 40 °C for 16 h.

### 4.4. Typical Procedure for L-Lactide Polymerization Without Solvent

Five 10 mL vials were loaded with 1.3 μmol of catalyst, 4,0 μmol of benzyl alcohol, and 2.0 mmol of technical grade L-LA and were immediately stirred at 175 °C (entry 7, Table 2). After the required polymerization time, the reaction mixture was quenched with an ice-water bath, and an aliquot of the crude material was sampled to determinate the monomer conversion via ^1^H-NMR spectroscopy.

### 4.5. Computational Details

All DFT calculations were performed at the GGA level with the Gaussian 09 set of programmes [64], using the BP86 functional of Becke and Perdew [65,66]. The electronic configuration of the molecular systems was described with the 6-31G(d) basis set for all the atoms. Geometry optimizations were performed without symmetry constraints, and all the obtained structures were validated as minima or transition states by using the vibrational frequency calculations.

To save computational resources, the phenyl substituents on the complexes were modelled with methyl groups, the growing polylactide chain was modelled with a methoxy group. The reported Gibbs free energies have been obtained by adding thermal corrections in gas phase to electronic energy in solvent (CPCM model) [67] computed via single-point calculation in toluene at the BP86-D3 level with the triple-ζ basis set of Ahlrichs (TZVP). The buried volume calculations were performed with the SambVca 2.1 package, software free of charge, developed by Cavallo et al. [56]. The radius of the sphere around the metal centre was set to 4.0 Å, while for the atoms, we adopted the Bondi radii scaled by 1.17, and a mesh of 0.1 Å was used to scan the sphere for buried voxels.

## Figures and Tables

**Table 1:** Styrene polymerization promoted by **1**/MAO.

Entry ^(a)^	Catalyst	Al/Ti	Temper. (°C)	Time (h)	Yield. (g)	TOF ^(b)^ (h^−1^)	*M*_n(expt)_ ^(c)^	*Đ* ^(c)^
1	**1**	500	60	3	0.089	28	6.6	1.7
2	**1**	500	60	6	0.130	21	6.7	1.7
3	**1**	500	60	18	0.172	9	8.2	3.2
4	**1**	500	80	18	0.252	13	6.1	1.6
5	**1**	250	80	18	0.241	6	6.4	1.4
6	**1**	1000	80	18	0.252	27	5.2	1.5

^(a)^ Polymerization conditions: solvent = toluene; Ti complex = 10 μmol [MAO] = 2.5–10 mM; styrene = 5.0 mmol [St] = 0.50 M; total volume = 10 mL. ^(b)^ TOF = turn over frequency; ^(c)^ experimental molecular weight *M*_n(expt)_ (kg mol^–1^) and dispersity (*Đ*) determined by GPC in THF using polystyrene standards.

**Table 2:** Ring-opening polymerization of L-lactide promoted by **2**/BzOH.

Entry ^(a)^	LA/2/BzOH	Temp.	Time (h)	Conv. ^(b)^ (%)	*M*_n(th)_ ^(c)^	*M*_n(expt)_ ^(d)^	*Đ* ^(d)^
1	150/1/0	100	16	23	-	-	-
2	150/1/1	100	16	71	15.4	12.3	1.24
3	150/1/1	100	20	87	18.8	12.5	1.32
4	150/1/2	100	16	90	9.7	7.7	1.24
5	150/1/2	100	20	95	10.3	8.9	1.23
6	150/1/3	100	16	93	6.7	5.3	1.19
7 ^(e)^	1500/1/3	175	1	81	58.3	10.2	2.22
8 ^(e)^	3000/1/6	175	1	83	59.8	11.6	2.26

^(a)^ All reactions were carried out in 2.0 mL of toluene as solvent, [cat]_0_ = 6.7 mM, [LA] = 1.0 M, [BzOH]_0_ = 6.7–20.0 mM. ^(b)^ Molecular conversion determined by ^1^H NMR spectroscopy (CDCl_3_, 298 K). ^(c)^ Calculated molecular weight using *M*_n(th)_ (kg mol^–1^) = (PM_Mon_ × ([Mon]_0_/[**2**]_0_) × conversion)/1000. ^(d)^ Experimental molecular weight *M*_n(expt)_ (kg mol^–1^) and dispersity (*Đ*) determined by GPC in THF using polystyrene standards and corrected using the factor 0.58. ^(e)^ Technical grade L-LA, no solvent.

## Data Availability

Data are contained within the article and Supplementary Materials.

## Supplementary Materials

The following supporting information can be downloaded at https://www.mdpi.com/article/10.3390/polym17192715/s1, NMR spectra, Cartesian coordinates of all the structures optimized in the computational analysis. Figure S1. 1H NMR spectrum of complex of [Ti{η5-C5H4(SiMeO2Ph7Si7O11-κO)}Cl2] (1); Figure S2:13C{1H} NMR spectrum of complex of [Ti{η5-C5H4(SiMeO2Ph7Si7O11-κO)}Cl2] (1); Figure S3: COSY 1H-1H spectrum of complex of [Ti{η5-C5H4(SiMeO2Ph7Si7O11-κO)}Cl2] (1); Figure S4: HMBC 1H- 29Si spectrum of complex of [Ti{η5-C5H4(SiMeO2Ph7Si7O11-κO)}Cl2] (1); Figure S5: 1H NMR spectra of the reaction mixture for the synthesis of at different times; Figure S6: 1H NMR of polystyrene; Figure S7: Effect of the temperature in the L-lactide polymerization; Figure S8: Plot of number-averaged molecular weights Mn(expt) vs monomer conversion; Figure S9: 1H NMR and 13C NMR spectra of PLA.; Figure S10: 1H NMR and 13C NMR spectra of oligomers of L-LA; Figure S11: MALDI-TOF spectrum of oligomers of L-LA obtained using 2/BnOH.; Table S1. Free energies of all the structures involved in the styrene polymerization.
