# Supplementary material for: Cyclopentadienyl–Silsesquioxane Titanium Complexes in the Polymerizations of Styrene and L-Lactide
Source: Polymers (Basel). 2025 Oct 9;17(19):2715. doi: 10.3390/polym17192715 (PMC12526836; doi:10.3390/polym17192715)
Supplement: Supplementary file 1 [file polymers-17-02715-s001.zip › polymers-3899963-supplementary.pdf]

# Cyclopentadienyl-Silsesquioxane Titanium Complexes in the Polymerizations of Styrene and Lactide

Joan Vinueza-Vaca, Shoaib Anwar, Salvatore Impemba, Ilaria Grimaldi, Gerardo Jiménez, Carmine Capacchione, Vanessa Tabernero,* Stefano Milione*

Department of Chemistry and Biology, University of Salerno, Via Giovanni Piano II, 84084 Fisciano, Salerno, Italy; Department of Organic and Inorganic Chemistry, Alcalà University, Campus Universitario, 28871, Alcala de Henares, Spain

*Contents*


| | *pag.* |
|---|---|
| **Experimental section** | *3* |
| ***Figure S1.*** $^{1}H$ NMR spectrum of complex of [Ti{$\eta^5$-$C_5H_4(SiMeO_2Ph_7Si_7O_{11}$-κO)}$Cl_2$] (**1**) | *4* |
| ***Figure S2:*** $^{13}C\{^{1}H\}$ NMR spectrum of complex of [Ti{$\eta^5$-$C_5H_4(SiMeO_2Ph_7Si_7O_{11}$-κO)}$Cl_2$] (**1**) | *4* |
| ***Figure S3:*** COSY $^{1}H$-$^{1}H$ spectrum of complex of [Ti{$\eta^5$-$C_5H_4(SiMeO_2Ph_7Si_7O_{11}$-κO)}$Cl_2$] (**1**) | *5* |
| ***Figure S4***: HMBC $^{1}H$-$^{29}Si$ spectrum of complex of [Ti{$\eta^5$-$C_5H_4(SiMeO_2Ph_7Si_7O_{11}$-κO)}$Cl_2$] (**1**) | *5* |
| ***Figure S5:*** $^{1}H$ NMR spectra of the reaction mixture for the synthesis of at different times | *6* |
| ***Figure S6:*** $^{1}H$ NMR of polystyrene | *7* |
| ***Figure S7:*** Effect of the temperature in the L-lactide polymerization | *7* |
| ***Figure S8:*** Plot of number-averaged molecular weights $M_{n(expt)}$ *vs* monomer conversion | *8* |
| ***Figure S9:*** $^{1}H$ NMR and $^{13}C$ NMR spectra of PLA. | *9* |
| ***Figure S10:*** $^{1}H$ NMR and $^{13}C$ NMR spectra of oligomers of L-LA | *10* |
| ***Figure S11:*** MALDI-TOF spectrum of oligomers of L-LA obtained using **2**/BnOH. | *11* |
| ***Table S1.*** Free energies of all the structures involved in the styrene polymerization. | *12* |
| Cartesian coordinates of all the structures optimized in the computational analysis | *13* |

**Experimental section**

**Materials and Methods.** All preparations and subsequent manipulations of air- and/or water-sensitive compounds were carried out under a dry nitrogen atmosphere using a Braun Labmaster drybox or standard Schlenk line techniques. Glassware and vials used in the polymerization were dried in an oven at 120 °C overnight and exposed three times to vacuum-nitrogen cycles. All solvents and reagents used were dried and purified before use. Toluene (Sigma-Aldrich, 99.5%), hexane (Sigma-Aldrich, 99%) were preliminarily dried over $CaCl_2$, while THF (Sigma-Aldrich, 99%) was preliminarily treated with KOH. Then, all solvents were purified by distillation from sodium under a nitrogen atmosphere. Ligands used for the synthesis of complexes were anhydrificated in vacuum with $P_2O_5$. Lactide was purified by crystallization from dry toluene and then stored over $P_2O_5$. All other chemicals were commercially available and used as received unless otherwise stated.

**Instruments and Measurements.** The NMR spectra were recorded on Bruker Avance 400 or Bruker Avance 600 spectrometer at 25 °C, unless otherwise stated. Deuterated solvents were purchased from Cambridge Isotope Laboratories, Inc., degassed and dried over activated 4Å molecular sieves prior to use. Chemical shifts (d) are listed as parts per million and coupling constants (J) in hertz. $^{1}$H NMR spectra are referenced using the residual solvent peak at d 7.16 for $C_6D_6$, d 7.27 for $CDCl_3$ and d 5.32 for $CD_2Cl_2$. $^{13}$C NMR spectra are referenced using the residual solvent peak at d 128.39 for $C_6D_6$, d 77.23 for $CDCl_3$ and d 53.84 for $CD_2Cl_2$. The molecular weights (Mn and Mw) and the molecular mass distribution (Mn/Mw) of polymer samples were measured by gel permeation chromatography (GPC) at 30 °C, using THF as solvent, flow rate of eluent 1 mL/min, and narrow polystyrene standards as reference. The measurements were performed on a Waters 1525 binary system equipped with a Waters 2414 RI detector using four Styragel columns (range 1000–1000000 Å). Every value was the average of two independent measurements. It was corrected using the factor of 0.58 for polylactide according to the literature.[1] High-Resolution Matrix Assisted Laser Desorption Ionization Analysis. In a general procedure of MALDI-TOF MS sample preparation a 1.0 mg of substance was dissolved in 1.0 mL of $CH_2Cl_2$. 4 µL of this solution was added in 45 µL of solution of dihydroxybenzoic acid 40 mM in $CH_2Cl_2$ as matrix agent.

[1] Biela, T.; Duda, A.; Penczek, S. Control of Mn, Mw/Mn, end-groups, and kinetics in living polymerization of cyclic esters. Macromol. Symp. 2002, 183, 1-10.

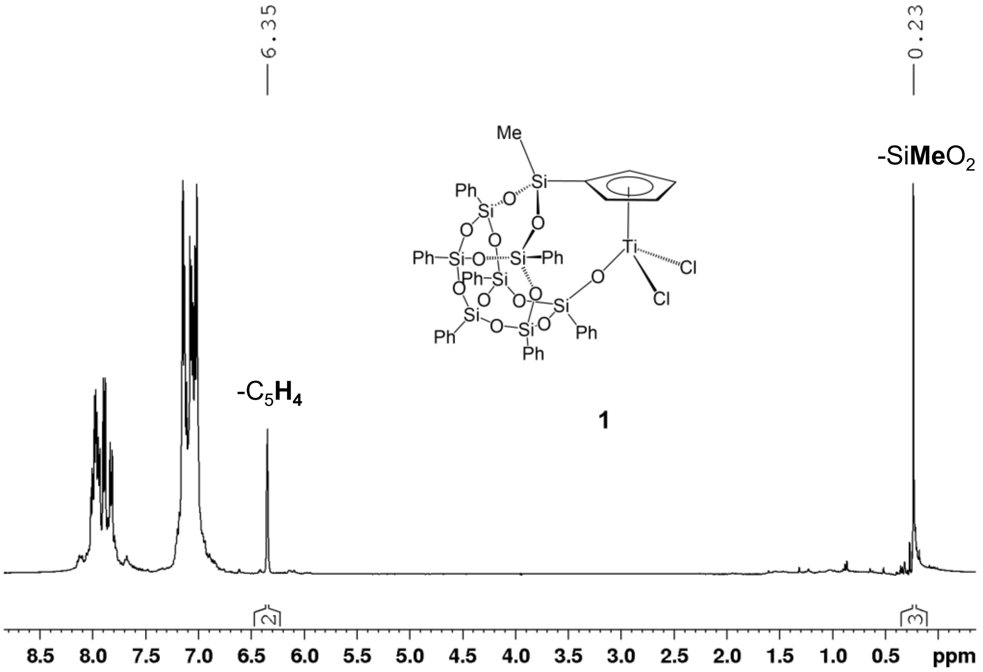


***Figure S1:*** $^{1}$H NMR spectrum of complex of [Ti{$\eta^5$-$C_5H_4(SiMeO_2Ph_7Si_7O_{11}$-κO)}$Cl_2$] (**1**)

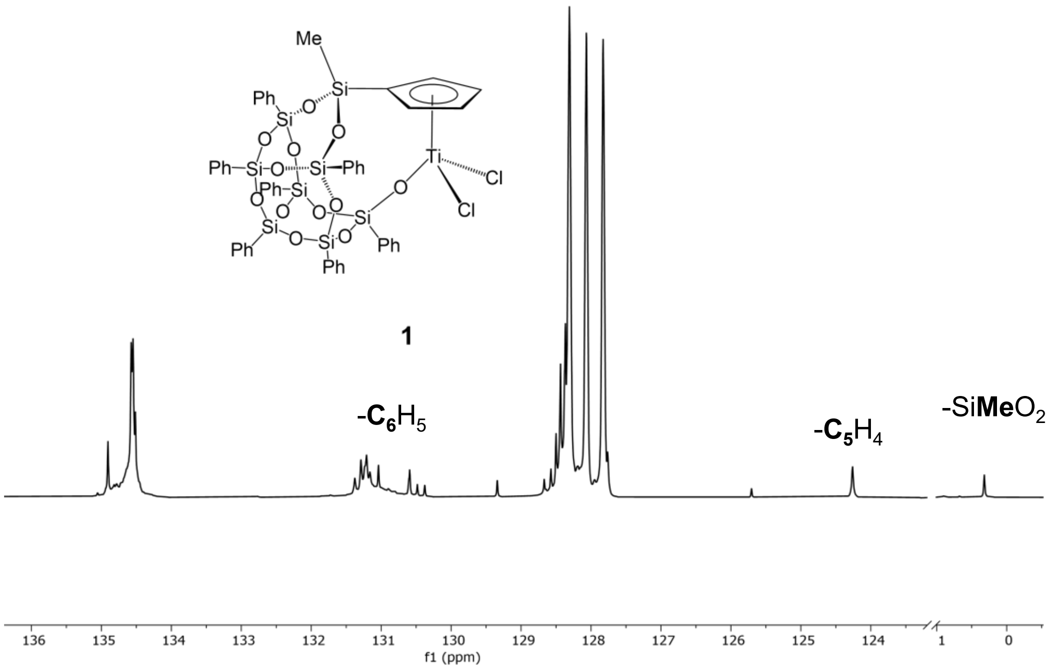


***Figure S2:*** $^{13}$C{$^{1}$H}-NMR spectrum of complex of [Ti{$\eta^5$-$C_5H_4(SiMeO_2Ph_7Si_7O_{11}$-κO)}$Cl_2$] (**1**)

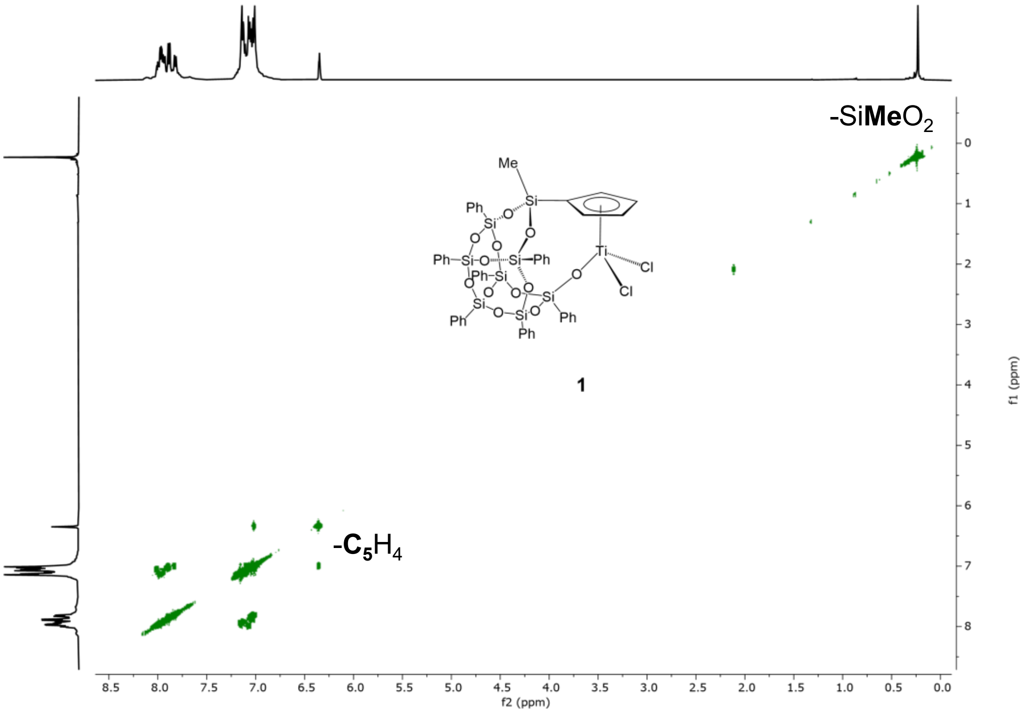


***Figure S3:*** COSY $^{1}H$-$^{1}H$ spectrum of complex of [Ti{$\eta^5$-$C_5H_4(SiMeO_2Ph_7Si_7O_{11}$-κO)}$Cl_2$] (**1**)

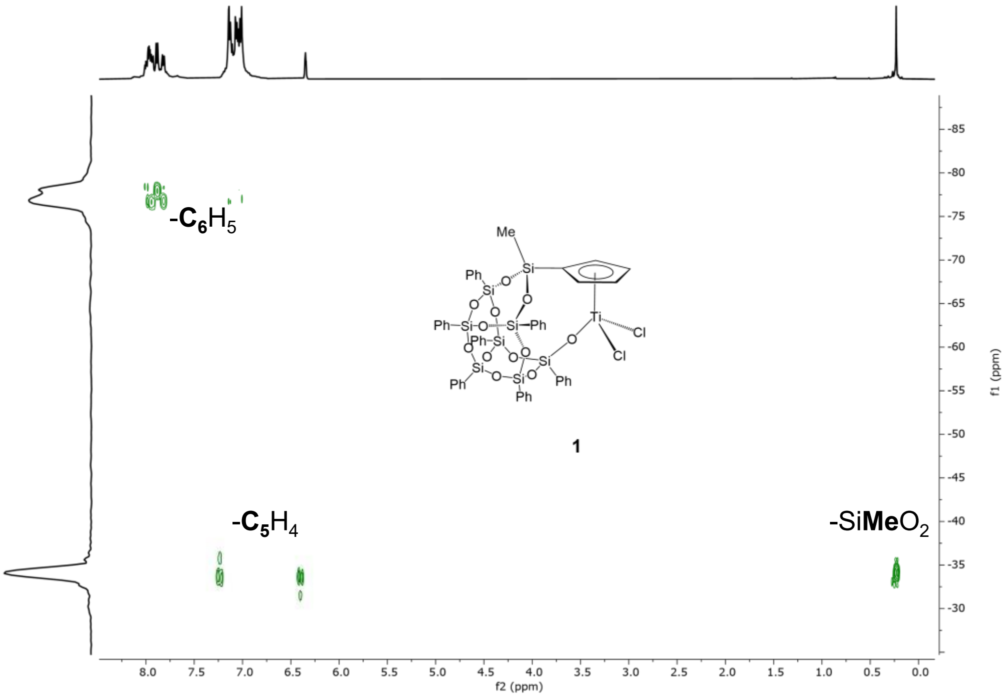


***Figure S4:*** HMBC $^{1}H$- $^{29}Si$ spectrum of complex of [Ti{$\eta^5$-$C_5H_4(SiMeO_2Ph_7Si_7O_{11}$-κO)}$Cl_2$] (**1**)

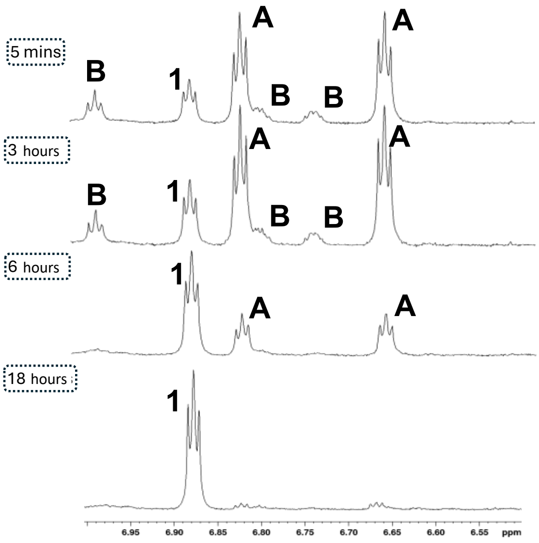


***Figure S5:*** $^{1}$H NMR spectra of the crude reaction mixture displaying the presence of the two intermediates A and B and target compound **1** at different reaction times.

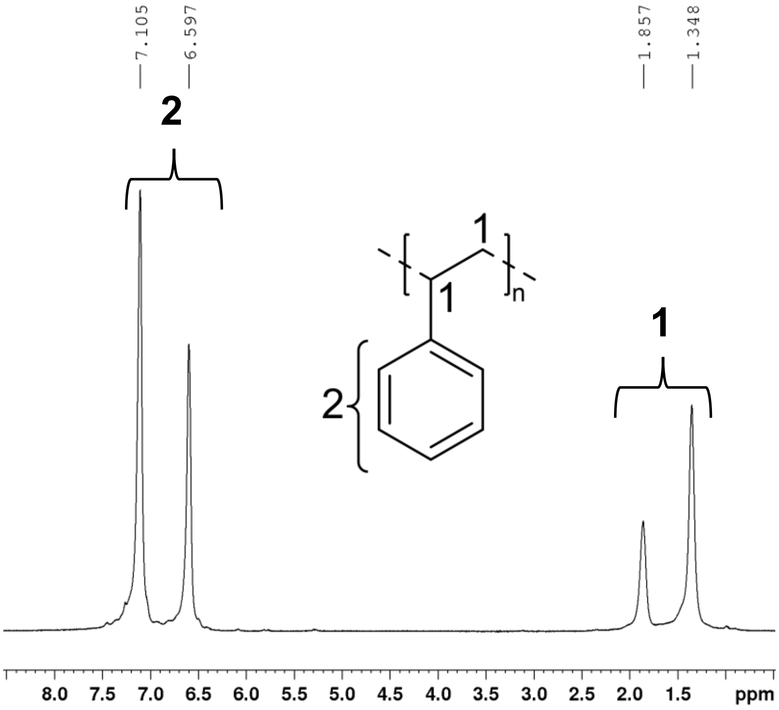


***Figure S6:*** $^{1}$H NMR of polystyrene obtained by **1**/MAO (Table 1, entry 4, CDCl3, 298 K)

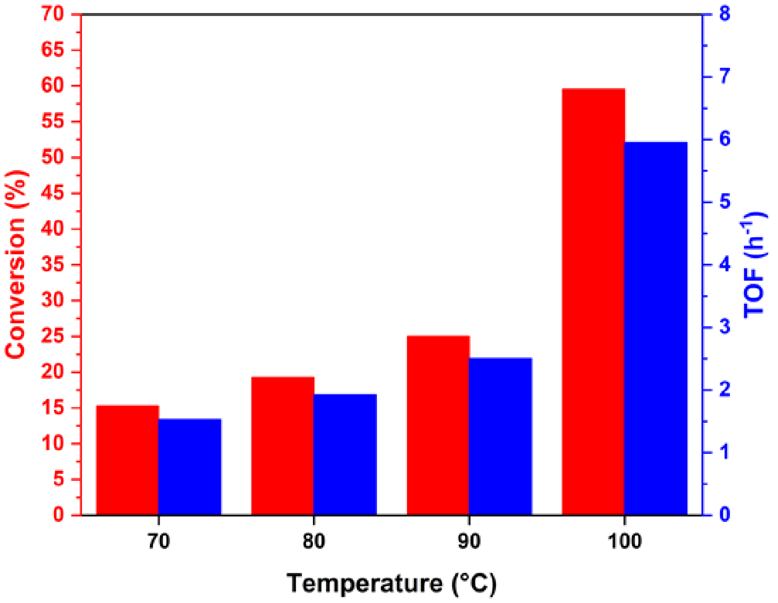


***Figure S7***. Effect of the temperature in L-lactide polymerization promoted by **2**/BzOH. Conditions: [*L*-LA]$_0$= 1.0 M; [*L*-LA]$_0$/[**2**]$_0$= 50, [BzOH]$_0$/[cat]$_0$= 1, time = 5 h, toluene (2.0 ml) as solvent.

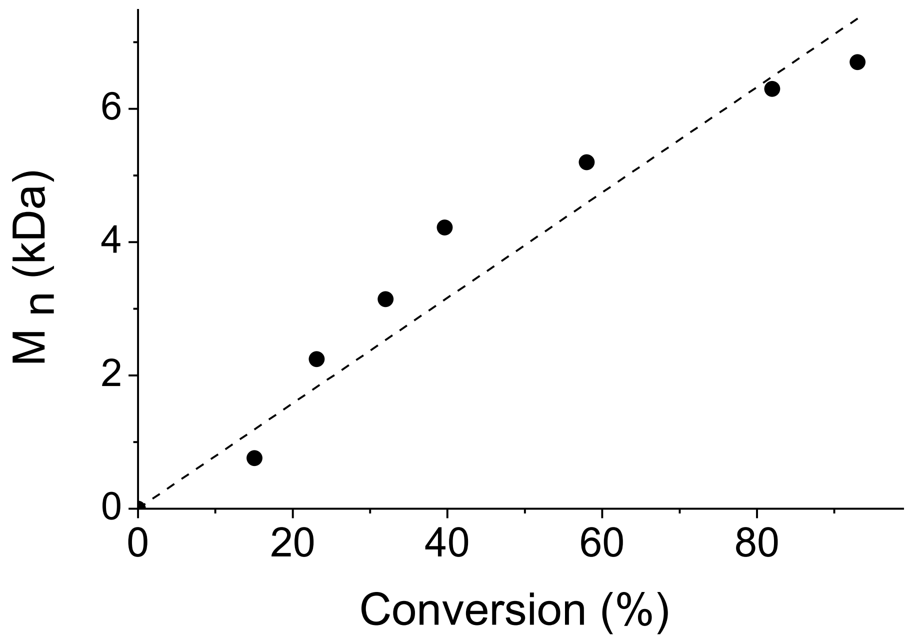


***Figure S8***. Plot of number-averaged molecular weights $M_n$(expt) vs monomer conversion (%) using **2**/BzOH. Conditions: $[LA]_0$= 1.0 M; $[LA]_0/[\mathbf{2}]_0/[BzOH]_0$= 150/1/3, T= 100 °C, toluene (2.0 ml) as solvent.

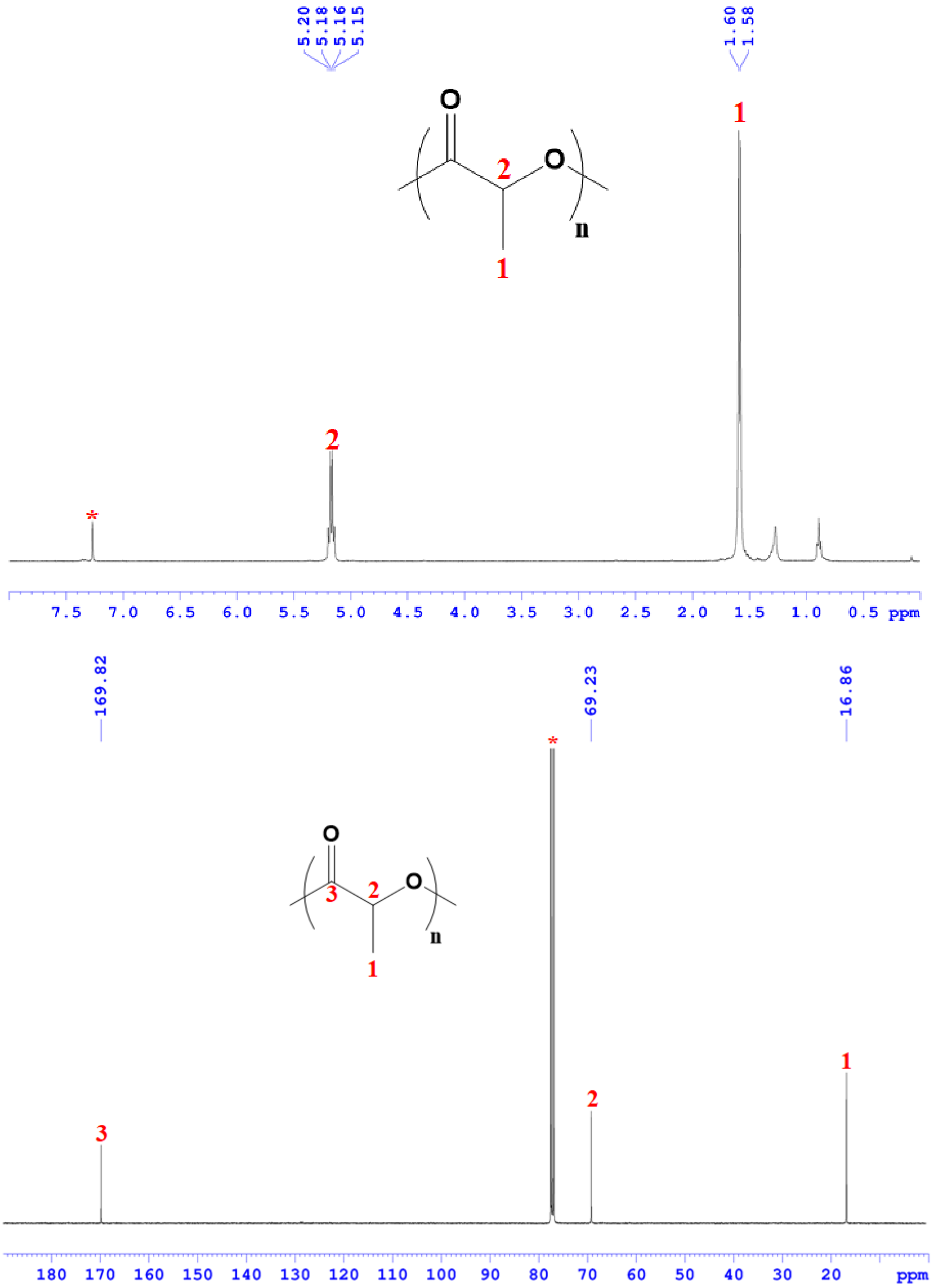


***Figure S9.*** $^{1}H$ NMR and $^{13}C$ NMR spectra (*$CDCl_3$, 298 K) of PLA obtained using **2**/BnOH as initiator after quenching with wet n-hexane. Conditions: $[L\text{-}LA]_0/[\mathbf{2}]_0 = 150$, $[BnOH]/[\mathbf{2}]_0 = 3$, Toluene 2 mL, T = 100 °C, t= 8 h.

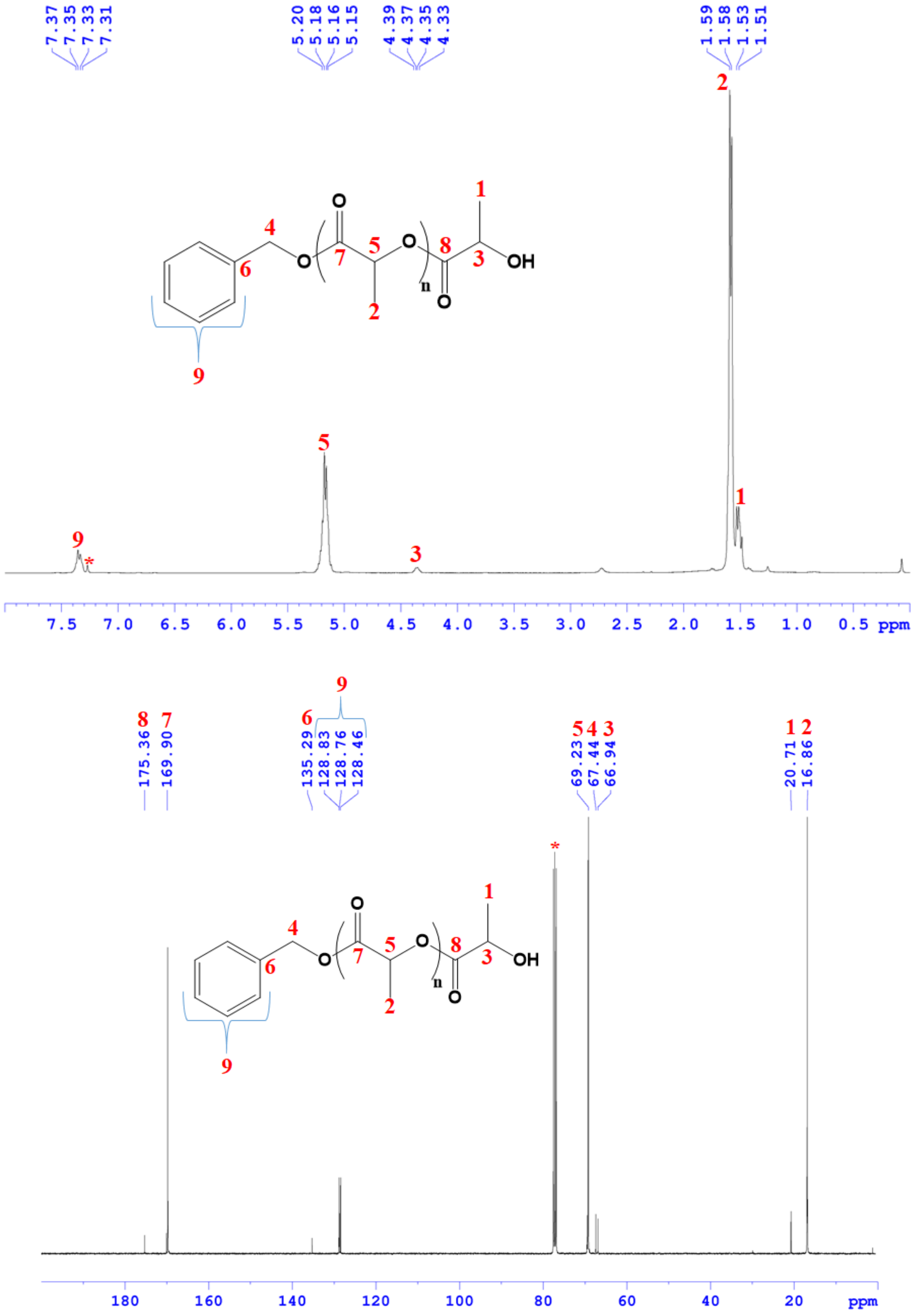


***Figure S10.*** $^{1}$H NMR and $^{13}$C NMR spectra (*$CDCl_3$, 298 K) of oligomers of L-LA obtained using **2**/BnOH as initiator after quenching with wet n-hexane. Conditions: $[L\text{-}LA]_0/[\mathbf{2}]_0 = 30$, $[BnOH]/[\mathbf{2}]_0 = 3$, Toluene 2 mL, T = 100 °C, t = 16 h.

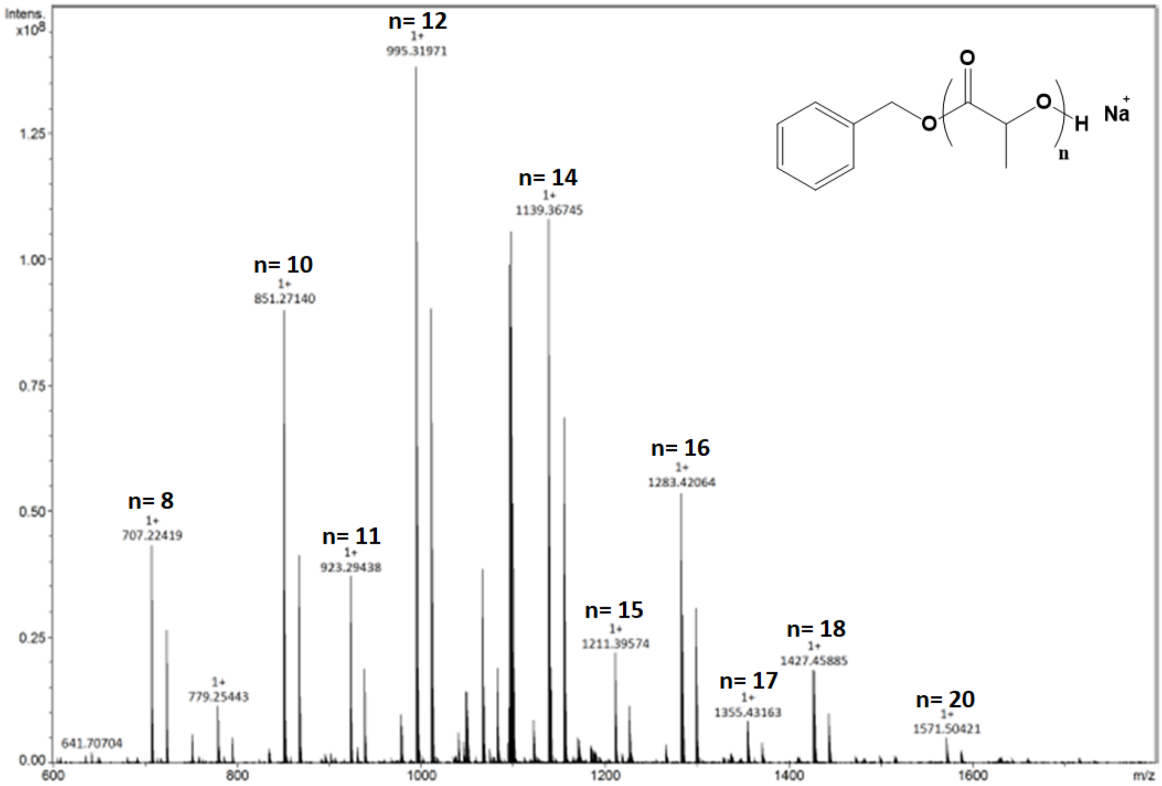


***Figure S11.*** MALDI-TOF spectrum of oligomers of L-LA obtained using **2**/BnOH as initiator.

***Table S1.*** Computed Energies and Gibbs free energies (in Hartrees) for all the optimized structures involved in the lactide polymerization (BP86-D3/TZVP/Lanl2DZ-CPCM(toluene)//BP86/6-31G(d)/Lanl2DZ).

| *label* | *E(solv)* | *G(solv)* |
|---|---|---|
| int0 | -4480,994024 | -4480,449547 |
| $intTS_{0-1}$ | -4480,955188 | -4480,407279 |
| int1 | -4480,980233 | -4480,429388 |
| int2 | -4480,980242 | -4480,429411 |
| $intTS_{2-3}$ | -4480,95598 | -4480,404389 |
| int3 | -4480,978897 | -4480,431752 |
| Int4 | -4480,996443 | -4480,452674 |

**XYZ Coordinates of Computed Structures**

**Int0**

| | | | |
|---|---|---|---|
| Ti | -1.32327800 | 1.24818400 | 0.61407100 |
| Si | 1.28139700 | 3.91304500 | -0.48116500 |
| Si | 3.48701900 | 1.70385500 | 0.21765500 |
| Si | 3.14264000 | -0.26903500 | -2.24491800 |
| Si | 0.05750100 | -3.00303500 | -0.31164300 |
| Si | -0.18605200 | -1.31944500 | 2.43161200 |
| Si | 2.90867100 | -0.73431500 | 2.19100500 |
| Si | 3.24451900 | -2.73391200 | -0.24676300 |
| Si | -0.05973800 | -0.36080800 | -2.02341500 |
| O | -0.25687900 | 0.73188300 | -0.77658500 |
| O | 2.57804100 | 3.08156100 | 0.18155800 |
| O | -0.85378200 | 0.13907000 | 1.98621200 |
| O | 3.54736500 | 1.03511900 | -1.30156900 |
| O | 2.77591600 | 0.65604100 | 1.29234800 |
| O | 3.58974900 | -1.67187600 | -1.47747300 |
| O | 1.51311800 | -0.26264900 | -2.55063100 |
| O | 1.69277300 | -3.28803800 | -0.40466700 |
| O | -0.37826200 | -1.90195900 | -1.48580100 |
| O | -0.30676700 | -2.40336700 | 1.18663000 |
| O | 1.41656500 | -1.08299100 | 2.82116600 |
| O | 3.44220200 | -1.97282100 | 1.21936100 |
| C | -0.27699200 | 3.43822500 | 0.46606200 |
| C | -1.64093200 | 3.56146800 | 0.03201600 |
| C | -2.50796700 | 3.28890100 | 1.13218900 |
| C | -1.69638600 | 2.97553800 | 2.25915600 |
| C | -0.33291600 | 3.05508200 | 1.84968500 |
| H | -3.59522500 | 3.24416900 | 1.07829500 |
| H | -2.04945300 | 2.64679400 | 3.23849200 |
| H | -1.97121900 | 3.78125600 | -0.98564000 |
| H | 0.53277500 | 2.81138900 | 2.46814300 |
| C | -0.86185900 | -4.59600400 | -0.59725900 |
| H | -0.55064700 | -5.35620700 | 0.13989100 |
| H | -1.94843200 | -4.43687500 | -0.48980800 |
| H | -0.65666600 | -4.99191600 | -1.60687800 |
| C | -1.20082600 | 0.04284000 | -3.44410200 |
| H | -2.24650700 | 0.04796900 | -3.09200100 |
| H | -0.96572600 | 1.03857400 | -3.85828100 |
| H | -1.10225500 | -0.70032100 | -4.25431700 |
| C | 4.42905600 | -4.17168500 | -0.33966700 |
| H | 4.32480700 | -4.70224300 | -1.30150900 |
| H | 5.47285900 | -3.82565900 | -0.24816400 |
| H | 4.23165500 | -4.89078900 | 0.47364500 |
| C | -1.07897000 | -1.99603200 | 3.92547000 |
| H | -2.14062700 | -2.17548900 | 3.68418700 |
| H | -0.63228800 | -2.95245800 | 4.24735100 |
| H | -1.02628800 | -1.28725300 | 4.76976400 |
| C | 4.10783600 | -0.53011900 | 3.60952600 |
| H | 4.16291500 | -1.45644000 | 4.20695000 |
| H | 5.12326900 | -0.29953300 | 3.24365400 |
| H | 3.78691600 | 0.28845100 | 4.27671400 |
| C | 5.22478800 | 2.14858300 | 0.75194000 |
| H | 5.86931800 | 1.25268900 | 0.78969100 |
| H | 5.67837100 | 2.86081300 | 0.04098700 |
| H | 5.22554400 | 2.61767300 | 1.75107200 |
| C | 4.05900600 | -0.14557400 | -3.86605300 |
| H | 5.14988700 | -0.15498700 | -3.70029400 |
| H | 3.80308100 | -0.99328700 | -4.52448000 |
| H | 3.79831100 | 0.78920700 | -4.39122300 |
| C | 1.60944200 | 5.74626800 | -0.15062900 |
| H | 0.77494700 | 6.37141100 | -0.51751200 |
| H | 1.72349300 | 5.93670400 | 0.93113300 |
| H | 2.53387400 | 6.08083500 | -0.65535700 |
| C | 1.10445200 | 3.59683100 | -2.33222700 |
| H | 1.97920500 | 3.99299300 | -2.87839500 |
| H | 1.02662700 | 2.51833000 | -2.54368100 |
| H | 0.20340400 | 4.09616700 | -2.73426200 |
| O | -2.96177000 | 0.56577800 | 0.12382000 |
| C | -4.25296200 | 0.51086000 | -0.33145700 |
| C | -5.18341300 | -0.10092800 | 0.75685300 |
| C | -4.75475700 | -1.66210600 | -1.37678500 |
| H | -6.20611100 | -0.18380200 | 0.33647600 |
| C | -4.46472600 | -2.26577000 | 0.00842200 |
| H | -5.86462500 | -1.67303800 | -1.48517100 |
| O | -4.30078700 | -0.29314600 | -1.51363200 |
| O | -4.71438800 | -1.44353700 | 1.06920500 |
| O | -4.09701900 | -3.41283400 | 0.19552900 |
| C | -5.21815700 | 0.67378400 | 2.06515800 |
| H | -4.20573500 | 0.74922700 | 2.49266500 |
| H | -5.61293600 | 1.68902500 | 1.89367200 |
| H | -5.87192300 | 0.15768000 | 2.78802400 |
| C | -4.12722800 | -2.48057500 | -2.49870000 |
| H | -3.03162800 | -2.50501900 | -2.38395300 |
| H | -4.50919700 | -3.51332700 | -2.46722000 |
| H | -4.37928600 | -2.03108400 | -3.47350100 |
| O | -4.63680800 | 1.83094700 | -0.68293500 |
| C | -5.89338900 | 1.97382700 | -1.36164400 |
| H | -5.97233200 | 1.26757500 | -2.20597600 |
| H | -6.75715400 | 1.83761000 | -0.67981000 |
| H | -5.90899900 | 3.00627400 | -1.74409300 |

**IntTS$_{0-1}$**

| | | | |
|---|---|---|---|
| Ti | 1.55508600 | 1.42680600 | 0.04280100 |
| Si | -1.70943600 | 3.59903500 | 0.21801500 |
| Si | -3.86946500 | 1.44790500 | -0.76285700 |
| Si | -3.45496300 | -0.34393400 | 1.78060300 |
| Si | -0.00292200 | -2.94270200 | 0.53249300 |
| Si | 0.30222300 | -1.04781700 | -1.95785400 |
| Si | -2.85400500 | -1.09659200 | -2.42586300 |
| Si | -3.15306900 | -2.97897200 | 0.18926000 |
| Si | -0.24018100 | -0.28506200 | 2.22146800 |
| O | 0.64963100 | 0.96898100 | 1.59693900 |
| O | -2.56886000 | 2.46004200 | -0.65310100 |
| O | 0.60671800 | 0.28383500 | -1.01143200 |
| O | -4.15299200 | 0.70852600 | 0.70407600 |
| O | -3.54746300 | 0.32651400 | -1.93742400 |
| O | -3.75810000 | -1.91315700 | 1.31929500 |
| O | -1.82081800 | -0.06781800 | 1.76648900 |
| O | -1.60143400 | -3.39298900 | 0.60947200 |
| O | 0.30558500 | -1.75694000 | 1.66060600 |
| O | 0.36086600 | -2.40630000 | -0.99419700 |
| O | -1.22027700 | -0.89805700 | -2.60646600 |
| O | -3.16975400 | -2.28272800 | -1.31129600 |
| C | 0.12218700 | 3.38278700 | -0.15404400 |
| C | 1.20750700 | 3.73995400 | 0.72389600 |
| C | 2.42200000 | 3.72187400 | -0.00600900 |
| C | 2.11882100 | 3.34651600 | -1.35128000 |

| | | | |
|---|---|---|---|
| C | 0.71471600 | 3.14571100 | -1.44075300 |
| H | 3.41566700 | 3.88018100 | 0.41428500 |
| H | 2.84174700 | 3.16209100 | -2.14611000 |
| H | 1.11404800 | 3.93005800 | 1.79493700 |
| H | 0.17532000 | 2.80500800 | -2.32569300 |
| C | 1.03256200 | -4.44601900 | 0.92117600 |
| H | 0.81147100 | -5.25718200 | 0.20629400 |
| H | 2.10828600 | -4.21454400 | 0.85153300 |
| H | 0.81985400 | -4.81783900 | 1.93813300 |
| C | -0.09059800 | -0.28698200 | 4.09131300 |
| H | 0.95174300 | -0.46848600 | 4.40574500 |
| H | -0.41048800 | 0.68064000 | 4.51565900 |
| H | -0.71808100 | -1.08179500 | 4.53166200 |
| C | -4.21890000 | -4.50974900 | 0.18660400 |
| H | -4.21166700 | -4.99181400 | 1.17920100 |
| H | -5.26294100 | -4.25659100 | -0.06541900 |
| H | -3.85263700 | -5.24100800 | -0.55410700 |
| C | 1.52538400 | -1.22503300 | -3.35557200 |
| H | 2.55321100 | -1.23057100 | -2.95869600 |
| H | 1.34887300 | -2.15990800 | -3.91519800 |
| H | 1.43374700 | -0.37803600 | -4.05715900 |
| C | -3.59196600 | -1.58390000 | -4.06996500 |
| H | -3.15953700 | -2.53478600 | -4.42578200 |
| H | -4.68514200 | -1.71089000 | -3.98884500 |
| H | -3.39117300 | -0.80964300 | -4.83034700 |
| C | -5.41791800 | 2.37978700 | -1.24295100 |
| H | -6.27520700 | 1.69096200 | -1.33635700 |
| H | -5.67383200 | 3.14031200 | -0.48465800 |
| H | -5.28167300 | 2.89203000 | -2.21093600 |
| C | -4.20626700 | -0.06954800 | 3.46883200 |
| H | -5.29692300 | -0.23575400 | 3.43671000 |
| H | -3.77808700 | -0.76683100 | 4.20955900 |
| H | -4.02716100 | 0.96142900 | 3.81908700 |
| C | -2.20489300 | 5.30808400 | -0.43285400 |
| H | -1.61121300 | 6.10610600 | 0.04919400 |
| H | -2.04021100 | 5.37683100 | -1.52272100 |
| H | -3.27306700 | 5.51360300 | -0.23620000 |
| C | -2.04497100 | 3.49780300 | 2.07375700 |
| H | -3.11363000 | 3.68125400 | 2.28676100 |
| H | -1.77821200 | 2.50482700 | 2.47018200 |
| H | -1.46104000 | 4.26031400 | 2.62153800 |
| O | 3.25038400 | 0.68613200 | -1.20986500 |
| C | 4.12923800 | 0.25346200 | -0.41191300 |
| C | 5.54340700 | 0.86960300 | -0.30944500 |
| C | 5.25014100 | -1.61900100 | 0.60920500 |
| H | 5.76737000 | 1.07482600 | 0.75347200 |
| C | 6.48735700 | -1.35325400 | -0.26133100 |
| H | 5.37689800 | -1.09151500 | 1.57684900 |
| O | 4.07473400 | -1.06764200 | -0.06627900 |
| O | 6.53179000 | -0.09872000 | -0.79955600 |
| O | 7.36242500 | -2.16680700 | -0.48972900 |
| C | 5.72390600 | 2.12298700 | -1.14888500 |
| H | 5.46032700 | 1.92330400 | -2.19955500 |
| H | 5.07504900 | 2.92614300 | -0.76864700 |
| H | 6.77337600 | 2.45547600 | -1.09383200 |
| C | 5.00606100 | -3.10052900 | 0.84278600 |
| H | 4.83000900 | -3.61672100 | -0.11462400 |
| H | 5.89454400 | -3.54724500 | 1.31619500 |
| H | 4.13340600 | -3.24162600 | 1.50024100 |
| O | 3.26420400 | 1.15881100 | 1.11395700 |
| C | 3.36349400 | 0.76592100 | 2.47004800 |
| H | 3.06605800 | -0.29352200 | 2.61346800 |
| H | 4.41172500 | 0.89600500 | 2.81584500 |
| H | 2.71203300 | 1.39281700 | 3.10539300 |

**Int1**

| | | | |
|---|---|---|---|
| Ti | -1.32327800 | 1.24818400 | 0.61407100 |
| Si | 1.28139700 | 3.91304500 | -0.48116500 |
| Si | 3.48701900 | 1.70385500 | 0.21765500 |
| Si | 3.14264000 | -0.26903500 | -2.24491800 |
| Si | 0.05750100 | -3.00303500 | -0.31164300 |
| Si | -0.18605200 | -1.31944500 | 2.43161200 |
| Si | 2.90867100 | -0.73431500 | 2.19100500 |
| Si | 3.24451900 | -2.73391200 | -0.24676300 |
| Si | -0.05973800 | -0.36080800 | -2.02341500 |
| O | -0.25687900 | 0.73188300 | -0.77658500 |
| O | 2.57804100 | 3.08156100 | 0.18155800 |
| O | -0.85378200 | 0.13907000 | 1.98621200 |
| O | 3.54736500 | 1.03511900 | -1.30156900 |
| O | 2.77591600 | 0.65604100 | 1.29234800 |
| O | 3.58974900 | -1.67187600 | -1.47747300 |
| O | 1.51311800 | -0.26264900 | -2.55063100 |
| O | 1.69277300 | -3.28803800 | -0.40466700 |
| O | -0.37826200 | -1.90195900 | -1.48580100 |
| O | -0.30676700 | -2.40336700 | 1.18663000 |
| O | 1.41656500 | -1.08299100 | 2.82116600 |
| O | 3.44220200 | -1.97282100 | 1.21936100 |
| C | -0.27699200 | 3.43822500 | 0.46606200 |
| C | -1.64093200 | 3.56146800 | 0.03201600 |
| C | -2.50796700 | 3.28890100 | 1.13218900 |
| C | -1.69638600 | 2.97553800 | 2.25915600 |
| C | -0.33291600 | 3.05508200 | 1.84968500 |
| H | -3.59522500 | 3.24416900 | 1.07829500 |
| H | -2.04945300 | 2.64679400 | 3.23849200 |
| H | -1.97121900 | 3.78125600 | -0.98564000 |
| H | 0.53277500 | 2.81138900 | 2.46814300 |
| C | -0.86185900 | -4.59600400 | -0.59725900 |
| H | -0.55064700 | -5.35620700 | 0.13989100 |
| H | -1.94843200 | -4.43687500 | -0.48980800 |
| H | -0.65666600 | -4.99191600 | -1.60687800 |
| C | -1.20082600 | 0.04284000 | -3.44410200 |
| H | -2.24650700 | 0.04796900 | -3.09200100 |
| H | -0.96572600 | 1.03857400 | -3.85828100 |
| H | -1.10225500 | -0.70032100 | -4.25431700 |
| C | 4.42905600 | -4.17168500 | -0.33966700 |
| H | 4.32480700 | -4.70224300 | -1.30150900 |
| H | 5.47285900 | -3.82565900 | -0.24816400 |
| H | 4.23165500 | -4.89078900 | 0.47364500 |
| C | -1.07897000 | -1.99603200 | 3.92547000 |
| H | -2.14062700 | -2.17548900 | 3.68418700 |
| H | -0.63228800 | -2.95245800 | 4.24735100 |
| H | -1.02628800 | -1.28725300 | 4.76976400 |
| C | 4.10783600 | -0.53011900 | 3.60952600 |
| H | 4.16291500 | -1.45644000 | 4.20695000 |
| H | 5.12326900 | -0.29953300 | 3.24365400 |
| H | 3.78691600 | 0.28845100 | 4.27671400 |
| C | 5.22478800 | 2.14858300 | 0.75194000 |
| H | 5.86931800 | 1.25268900 | 0.78969100 |
| H | 5.67837100 | 2.86081300 | 0.04098700 |
| H | 5.22554400 | 2.61767300 | 1.75107200 |
| C | 4.05900600 | -0.14557400 | -3.86605300 |

| | | | |
|---|---|---|---|
| H | 5.14988700 | -0.15498700 | -3.70029400 |
| H | 3.80308100 | -0.99328700 | -4.52448000 |
| H | 3.79831100 | 0.78920700 | -4.39122300 |
| C | 1.60944200 | 5.74626800 | -0.15062900 |
| H | 0.77494700 | 6.37141100 | -0.51751200 |
| H | 1.72349300 | 5.93670400 | 0.93113300 |
| H | 2.53387400 | 6.08083500 | -0.65535700 |
| C | 1.10445200 | 3.59683100 | -2.33222700 |
| H | 1.97920500 | 3.99299300 | -2.87839500 |
| H | 1.02662700 | 2.51833000 | -2.54368100 |
| H | 0.20340400 | 4.09616700 | -2.73426200 |
| O | -2.96177000 | 0.56577800 | 0.12382000 |
| C | -4.25296200 | 0.51086000 | -0.33145700 |
| C | -5.18341300 | -0.10092800 | 0.75685300 |
| C | -4.75475700 | -1.66210600 | -1.37678500 |
| H | -6.20611100 | -0.18380200 | 0.33647600 |
| C | -4.46472600 | -2.26577000 | 0.00842200 |
| H | -5.86462500 | -1.67303800 | -1.48517100 |
| O | -4.30078700 | -0.29314600 | -1.51363200 |
| O | -4.71438800 | -1.44353700 | 1.06920500 |
| O | -4.09701900 | -3.41283400 | 0.19552900 |
| C | -5.21815700 | 0.67378400 | 2.06515800 |
| H | -4.20573500 | 0.74922700 | 2.49266500 |
| H | -5.61293600 | 1.68902500 | 1.89367200 |
| H | -5.87192300 | 0.15768000 | 2.78802400 |
| C | -4.12722800 | -2.48057500 | -2.49870000 |
| H | -3.03162800 | -2.50501900 | -2.38395300 |
| H | -4.50919700 | -3.51332700 | -2.46722000 |
| H | -4.37928600 | -2.03108400 | -3.47350100 |
| O | -4.63680800 | 1.83094700 | -0.68293500 |
| C | -5.89338900 | 1.97382700 | -1.36164400 |
| H | -5.97233200 | 1.26757500 | -2.20597600 |
| H | -6.75715400 | 1.83761000 | -0.67981000 |
| H | -5.90899900 | 3.00627400 | -1.74409300 |

**Int2**

| | | | |
|---|---|---|---|
| Ti | 1.42995300 | 0.91087300 | -0.61472200 |
| Si | -0.82635400 | 3.94769600 | 0.20832800 |
| Si | -3.32423400 | 1.99277000 | -0.35408800 |
| Si | -3.15147500 | 0.17926100 | 2.27237600 |
| Si | -0.52772800 | -3.05894700 | 0.45171200 |
| Si | -0.15173900 | -1.53621700 | -2.29072300 |
| Si | -3.18599700 | -0.65197400 | -2.11477300 |
| Si | -3.65256000 | -2.39827900 | 0.44997000 |
| Si | 0.00179800 | -0.37856500 | 2.15402800 |
| O | 0.56075900 | 0.65138200 | 0.96740700 |
| O | -2.19031100 | 3.18860400 | -0.40119000 |
| O | 0.50411300 | -0.07846600 | -1.84087400 |
| O | -3.46907200 | 1.39160000 | 1.18580400 |
| O | -2.82884800 | 0.79952900 | -1.39720900 |
| O | -3.67773900 | -1.27687800 | 1.67255300 |
| O | -1.52517300 | 0.11970600 | 2.58455000 |
| O | -2.18463900 | -3.16733300 | 0.41639700 |
| O | -0.06534400 | -1.92777000 | 1.56733200 |
| O | 0.03134000 | -2.64507700 | -1.05988100 |
| O | -1.76584500 | -1.33034700 | -2.63576900 |
| O | -3.93185600 | -1.64699600 | -1.00935400 |
| C | 0.70822300 | 3.22518900 | -0.61525700 |
| C | 2.03635000 | 3.17614600 | -0.06366800 |
| C | 2.94432300 | 2.73094300 | -1.06628300 |
| C | 2.19420000 | 2.49020700 | -2.25650700 |
| C | 0.83083100 | 2.79181400 | -1.98052000 |
| H | 4.00595300 | 2.53098400 | -0.92427900 |
| H | 2.58564800 | 2.06068600 | -3.18013000 |
| H | 2.29904900 | 3.39338700 | 0.97410300 |
| H | -0.00420200 | 2.64751100 | -2.66820700 |
| C | 0.18473900 | -4.71748200 | 0.92131300 |
| H | -0.10078100 | -5.48923300 | 0.18608800 |
| H | 1.28614100 | -4.67059300 | 0.96648900 |
| H | -0.18488700 | -5.03105700 | 1.91256400 |
| C | 1.08963400 | -0.32419100 | 3.66826200 |
| H | 2.11442300 | -0.66914700 | 3.44950300 |
| H | 1.14618400 | 0.70385700 | 4.06657100 |
| H | 0.67206400 | -0.97281800 | 4.45823100 |
| C | -4.99934100 | -3.65456100 | 0.74480600 |
| H | -4.86401400 | -4.15157000 | 1.72061700 |
| H | -5.99174100 | -3.17209400 | 0.73876600 |
| H | -4.98818300 | -4.42910000 | -0.04105300 |
| C | 0.70092300 | -2.19516400 | -3.81767300 |
| H | 1.77932400 | -2.33188700 | -3.62915700 |
| H | 0.27335900 | -3.16698700 | -4.11884100 |
| H | 0.58572100 | -1.49009700 | -4.65884300 |
| C | -4.31901100 | -0.44204500 | -3.58496400 |
| H | -4.49306600 | -1.41224600 | -4.08137800 |
| H | -5.29813200 | -0.03496800 | -3.27908000 |
| H | -3.87434200 | 0.24653300 | -4.32402800 |
| C | -4.97494000 | 2.70395200 | -0.87562400 |
| H | -5.75666500 | 1.92419000 | -0.88912400 |
| H | -5.29783400 | 3.49332200 | -0.17481800 |
| H | -4.91242300 | 3.14857000 | -1.88393700 |
| C | -4.06053400 | 0.54143800 | 3.86145200 |
| H | -5.14780300 | 0.60776500 | 3.68514500 |
| H | -3.87991500 | -0.25498200 | 4.60362800 |
| H | -3.72277500 | 1.49826100 | 4.29512800 |
| C | -0.95850800 | 5.76010900 | -0.31798400 |
| H | -0.06508200 | 6.33131400 | -0.00637500 |
| H | -1.04932400 | 5.84735100 | -1.41514300 |
| H | -1.84510400 | 6.23832900 | 0.13655100 |
| C | -0.72241900 | 3.80431800 | 2.08510900 |
| H | -1.57964600 | 4.31376800 | 2.56087700 |
| H | -0.72856200 | 2.74889800 | 2.40266500 |
| H | 0.20102800 | 4.27696100 | 2.46790200 |
| O | 2.95375500 | -0.11472300 | -0.35938700 |
| C | 4.28106100 | -0.31282500 | -0.64836000 |
| C | 4.66871000 | -1.77653200 | -0.29337100 |
| C | 5.69769600 | 0.09528700 | 1.32157500 |
| H | 5.75683400 | -1.90360500 | -0.46505400 |
| C | 4.87201000 | -1.02596100 | 1.97766800 |
| H | 6.68531200 | -0.35784200 | 1.07097400 |
| O | 5.10476500 | 0.59451300 | 0.09837000 |
| O | 4.40933000 | -1.98572900 | 1.12607900 |
| O | 4.66830200 | -1.11303200 | 3.17689100 |
| C | 3.88275400 | -2.82852000 | -1.05875700 |
| H | 2.80104400 | -2.69219200 | -0.90140800 |
| H | 4.09641200 | -2.74243900 | -2.13734000 |
| H | 4.17478800 | -3.83680100 | -0.72020300 |
| C | 5.90627900 | 1.27701500 | 2.26095600 |
| H | 4.93510100 | 1.70934200 | 2.55091200 |
| H | 6.42521900 | 0.94564200 | 3.17423800 |
| H | 6.51117900 | 2.05087400 | 1.75974100 |
| O | 4.45865300 | -0.02002500 | -2.02327900 |

| | | | |
|---|---|---|---|
| C | 5.80326900 | -0.07839500 | -2.52194500 |
| H | 6.50294800 | 0.44633800 | -1.84790900 |
| H | 6.14561400 | -1.12128300 | -2.67710300 |
| H | 5.78319400 | 0.43129400 | -3.49789700 |

**IntTS$_{2-3}$**

| | | | |
|---|---|---|---|
| Ti | 1.88294800 | 0.78718500 | -0.35608100 |
| Si | -0.61858700 | 3.79300000 | 0.36357400 |
| Si | -3.27804100 | 2.12840100 | -0.59469300 |
| Si | -3.25232100 | 0.28757500 | 1.85884100 |
| Si | -0.63701100 | -3.17887500 | 0.48387000 |
| Si | 0.12877300 | -1.64917700 | -2.07657900 |
| Si | -2.83694500 | -0.53368300 | -2.28681500 |
| Si | -3.65725700 | -2.34200800 | 0.26544200 |
| Si | -0.13260300 | -0.36842200 | 2.01599400 |
| O | 1.03232700 | 0.56690400 | 1.26990700 |
| O | -2.05147400 | 3.15044700 | -0.20093900 |
| O | 0.83270600 | -0.46195400 | -1.16432400 |
| O | -3.87875600 | 1.37638300 | 0.76658900 |
| O | -2.68766600 | 1.00141900 | -1.66748300 |
| O | -3.80206200 | -1.24327100 | 1.51185000 |
| O | -1.60184800 | 0.35516300 | 1.70447100 |
| O | -2.30225300 | -3.27041100 | 0.50412000 |
| O | -0.14167200 | -1.91177900 | 1.42460800 |
| O | -0.13557700 | -2.96638500 | -1.09045500 |
| O | -1.32323800 | -1.08501500 | -2.67056800 |
| O | -3.56374500 | -1.52850500 | -1.17820700 |
| C | 0.80934100 | 2.97379000 | -0.55462900 |
| C | 2.21473500 | 3.19783600 | -0.34906400 |
| C | 2.93414000 | 2.67660400 | -1.45825300 |
| C | 1.99177800 | 2.12304700 | -2.37392100 |
| C | 0.69498900 | 2.29931000 | -1.82184600 |
| H | 4.01841300 | 2.61027300 | -1.54639600 |
| H | 2.23557600 | 1.56875800 | -3.28107200 |
| H | 2.66525900 | 3.63825400 | 0.54252300 |
| H | -0.24096600 | 1.92923000 | -2.24347800 |
| C | 0.06635100 | -4.77321500 | 1.14505700 |
| H | -0.20432400 | -5.62258400 | 0.49470800 |
| H | 1.16634900 | -4.70960500 | 1.20059300 |
| H | -0.31684100 | -4.97735000 | 2.15945000 |
| C | 0.16673900 | -0.43139300 | 3.86178900 |
| H | 1.14467100 | -0.89622300 | 4.06852200 |
| H | 0.14922900 | 0.57767900 | 4.30983200 |
| H | -0.61648000 | -1.03755500 | 4.35116700 |
| C | -5.15834500 | -3.44936900 | 0.25941800 |
| H | -5.25193000 | -3.98295600 | 1.22079200 |
| H | -6.07718100 | -2.85881300 | 0.10249200 |
| H | -5.08779700 | -4.20073600 | -0.54556700 |
| C | 1.18345300 | -2.17060800 | -3.52698500 |
| H | 2.20766100 | -2.39606800 | -3.18758600 |
| H | 0.76272500 | -3.06538800 | -4.01742700 |
| H | 1.24275300 | -1.36107600 | -4.27467900 |
| C | -3.86788500 | -0.51016300 | -3.84553100 |
| H | -3.93733000 | -1.52144300 | -4.28190800 |
| H | -4.89233800 | -0.15612100 | -3.63706600 |
| H | -3.41963600 | 0.15894100 | -4.60020100 |
| C | -4.67211800 | 3.10559200 | -1.36915500 |
| H | -5.52282200 | 2.44813400 | -1.61954900 |
| H | -5.03597400 | 3.88271400 | -0.67497200 |
| H | -4.33635800 | 3.60322700 | -2.29526100 |
| C | -3.82918100 | 0.74055700 | 3.57726700 |
| H | -4.93181500 | 0.76613300 | 3.61940900 |
| H | -3.47665600 | 0.00656300 | 4.32221300 |
| H | -3.45160300 | 1.73640700 | 3.86589100 |
| C | -0.60960800 | 5.61705500 | -0.14687200 |
| H | 0.32747000 | 6.11394000 | 0.16463400 |
| H | -0.69908500 | 5.72272400 | -1.24243700 |
| H | -1.45306500 | 6.15900500 | 0.31848800 |
| C | -0.47667100 | 3.66428900 | 2.23936000 |
| H | -1.27597000 | 4.24849800 | 2.73027100 |
| H | -0.55353400 | 2.61708900 | 2.56984800 |
| H | 0.49423400 | 4.06523000 | 2.58460000 |
| O | 3.39934300 | -0.39769000 | -1.60471100 |
| C | 4.49516600 | -0.33884100 | -1.00642300 |
| C | 4.98088800 | -1.49164900 | -0.10759900 |
| C | 4.04753200 | 0.24582400 | 1.86698800 |
| H | 5.84914200 | -1.17686200 | 0.49714200 |
| C | 3.63219900 | -1.23590900 | 1.91232400 |
| H | 5.16384500 | 0.24461000 | 1.93152200 |
| O | 3.69852200 | 0.80714600 | 0.61296900 |
| O | 3.94908300 | -1.97184800 | 0.77922500 |
| O | 3.04456800 | -1.79645000 | 2.81905700 |
| C | 5.37229800 | -2.67955800 | -0.99695600 |
| H | 4.49827800 | -3.00469500 | -1.58379900 |
| H | 6.18143000 | -2.39738400 | -1.69290200 |
| H | 5.71533800 | -3.51638400 | -0.36715800 |
| C | 3.54046100 | 1.05007800 | 3.06624100 |
| H | 2.44546100 | 1.12877900 | 3.03586500 |
| H | 3.84526700 | 0.56628700 | 4.00999200 |
| H | 3.97524200 | 2.06362500 | 3.02735500 |
| O | 5.43253200 | 0.49454500 | -1.53766800 |
| C | 6.66629100 | 0.76514200 | -0.83771800 |
| H | 6.45561400 | 1.14190700 | 0.17696600 |
| H | 7.32355300 | -0.12156800 | -0.79790400 |
| H | 7.16357900 | 1.55129300 | -1.42450200 |

**Int3**

| | | | |
|---|---|---|---|
| Ti | 1.54072500 | 1.45378500 | -0.26335400 |
| Si | -1.70124800 | 3.66240400 | -0.07452600 |
| Si | -3.88993300 | 1.35160900 | -0.61686200 |
| Si | -3.29504100 | -0.31576400 | 2.02453600 |
| Si | 0.17021200 | -2.92489800 | 0.59564500 |
| Si | 0.40662100 | -1.25418700 | -2.02853200 |
| Si | -2.75516300 | -1.16445700 | -2.20814900 |
| Si | -2.92220200 | -3.03109400 | 0.41833500 |
| Si | -0.06090900 | -0.09774500 | 2.18754400 |
| O | 0.57429700 | 1.13891100 | 1.25624900 |
| O | -2.64179700 | 2.42917700 | -0.69823000 |
| O | 1.21303500 | 0.01836900 | -1.30721000 |
| O | -3.98913800 | 0.69427000 | 0.90587800 |
| O | -3.62505200 | 0.17029400 | -1.75014600 |
| O | -3.41982900 | -1.89245700 | 1.52369100 |
| O | -1.70658000 | 0.11093200 | 2.21009500 |
| O | -1.37725700 | -3.52421200 | 0.78190700 |
| O | 0.32305600 | -1.55146900 | 1.50958100 |
| O | 0.38613100 | -2.56091400 | -1.01280000 |
| O | -1.15370300 | -0.73481100 | -2.29820800 |
| O | -2.96831700 | -2.38686000 | -1.11025200 |
| C | 0.05455700 | 3.37857100 | -0.69440400 |
| C | 1.27181100 | 3.87657000 | -0.10961000 |

| | | | |
|---|---|---|---|
| C | 2.35521500 | 3.61750300 | -0.99503500 |
| C | 1.83183800 | 2.93619100 | -2.13519400 |
| C | 0.42453900 | 2.79499600 | -1.94971200 |
| H | 3.40714700 | 3.84282100 | -0.80904500 |
| H | 2.41006100 | 2.54336000 | -2.97424900 |
| H | 1.35318800 | 4.34911100 | 0.87178700 |
| H | -0.25789700 | 2.27028900 | -2.62069400 |
| C | 1.41229300 | -4.20005600 | 1.13501100 |
| H | 1.29363400 | -5.13102400 | 0.55448800 |
| H | 2.42882200 | -3.80854000 | 0.96158300 |
| H | 1.29877000 | -4.43954500 | 2.20600200 |
| C | 0.61617700 | 0.00118700 | 3.92542600 |
| H | 1.71509300 | -0.10246600 | 3.92363800 |
| H | 0.35339700 | 0.96482800 | 4.39610900 |
| H | 0.19480400 | -0.80880600 | 4.54655300 |
| C | -4.06402600 | -4.50336900 | 0.51059500 |
| H | -4.03180400 | -4.95877200 | 1.51521300 |
| H | -5.10549800 | -4.20588500 | 0.29978900 |
| H | -3.76813700 | -5.27151300 | -0.22426400 |
| C | 1.23893600 | -1.72291500 | -3.63216400 |
| H | 2.30284800 | -1.94059700 | -3.44158300 |
| H | 0.76814200 | -2.62035500 | -4.06976300 |
| H | 1.17112900 | -0.90293800 | -4.36816500 |
| C | -3.38197800 | -1.72807300 | -3.87548200 |
| H | -2.85176200 | -2.63651600 | -4.20992800 |
| H | -4.46042900 | -1.95822700 | -3.83122400 |
| H | -3.22984800 | -0.94142200 | -4.63453400 |
| C | -5.52246500 | 2.17685300 | -1.00415400 |
| H | -6.34581400 | 1.44223300 | -0.98108400 |
| H | -5.75100600 | 2.96761500 | -0.26852800 |
| H | -5.50053200 | 2.63663100 | -2.00720100 |
| C | -4.20631900 | -0.12755800 | 3.64341700 |
| H | -5.27454200 | -0.37631800 | 3.52153900 |
| H | -3.78333200 | -0.79949000 | 4.40996000 |
| H | -4.13479400 | 0.90858300 | 4.01627400 |
| C | -2.30925300 | 5.29328300 | -0.82189800 |
| H | -1.66692800 | 6.13627400 | -0.50727800 |
| H | -2.29539100 | 5.25261600 | -1.92543700 |
| H | -3.34289500 | 5.51782900 | -0.50108500 |
| C | -1.77691700 | 3.74095700 | 1.80793500 |
| H | -2.79046200 | 4.02317100 | 2.14616200 |
| H | -1.52165100 | 2.76401200 | 2.24894900 |
| H | -1.07254000 | 4.49657400 | 2.20256700 |
| O | 3.70847400 | -2.20377200 | -0.73011300 |
| C | 4.48185900 | -1.30627300 | -1.00282100 |
| C | 5.55073200 | -0.79320600 | -0.01527400 |
| C | 4.16269200 | 1.35985600 | 1.36484900 |
| H | 5.78436100 | 0.26803400 | -0.19255900 |
| C | 4.43176900 | -0.00991500 | 2.04559300 |
| H | 5.12682500 | 1.74859200 | 0.96213600 |
| O | 3.28606100 | 1.12628400 | 0.27645800 |
| O | 5.08794400 | -0.98416500 | 1.33745100 |
| O | 4.09373500 | -0.26447800 | 3.18852600 |
| C | 6.82100700 | -1.64649100 | -0.15394700 |
| H | 6.58031500 | -2.70033300 | 0.06145600 |
| H | 7.23234800 | -1.58229800 | -1.17636700 |
| H | 7.58642100 | -1.30127200 | 0.56061300 |
| C | 3.63544500 | 2.36916500 | 2.38711200 |
| H | 2.66997400 | 2.03633400 | 2.79752700 |
| H | 4.35046700 | 2.47805400 | 3.22019800 |
| H | 3.50478300 | 3.35095200 | 1.90166000 |
| O | 4.51013000 | -0.82125900 | -2.28420300 |
| C | 5.25713100 | 0.36536800 | -2.62061500 |
| H | 4.90265000 | 1.23169200 | -2.03562500 |
| H | 6.34497000 | 0.22644100 | -2.48237400 |
| H | 5.05667600 | 0.53827300 | -3.68844400 |

**Int4**

| | | | |
|---|---|---|---|
| Ti | 1.09546900 | 0.30269700 | -0.90503400 |
| Si | -0.24166900 | 3.79133200 | -0.04958200 |
| Si | -3.13740500 | 2.40536900 | -0.11867900 |
| Si | -3.09631300 | 0.62556200 | 2.52339900 |
| Si | -1.43338800 | -3.08110700 | 0.49516100 |
| Si | -1.12448300 | -1.70398800 | -2.36513700 |
| Si | -3.85996400 | -0.22281900 | -1.82922700 |
| Si | -4.35311900 | -1.78609800 | 0.84240700 |
| Si | -0.19978500 | -0.56940100 | 1.99227300 |
| O | 0.17100900 | 0.38782900 | 0.68353100 |
| O | -1.79325000 | 3.29962200 | -0.45358200 |
| O | -0.09769100 | -0.43057000 | -2.10041800 |
| O | -3.10609700 | 1.90124900 | 1.46355800 |
| O | -3.14798300 | 1.10340700 | -1.14220200 |
| O | -4.07910200 | -0.58740200 | 1.95648300 |
| O | -1.54921800 | 0.06546700 | 2.73253600 |
| O | -3.04657200 | -2.80222500 | 0.76972700 |
| O | -0.51839100 | -2.12668900 | 1.49799900 |
| O | -1.07197000 | -2.77758400 | -1.09373200 |
| O | -2.67937000 | -1.13584200 | -2.54996400 |
| O | -4.63455800 | -1.10899100 | -0.65115800 |
| C | 1.01791000 | 2.72573200 | -0.96095500 |
| C | 2.31351600 | 2.32780300 | -0.47648100 |
| C | 3.01075000 | 1.64382300 | -1.51267400 |
| C | 2.15493100 | 1.59512900 | -2.65398600 |
| C | 0.93898900 | 2.25521800 | -2.31492900 |
| H | 4.00697600 | 1.20827300 | -1.42465400 |
| H | 2.36408000 | 1.08418200 | -3.59635800 |
| H | 2.68607800 | 2.46599400 | 0.54029900 |
| H | 0.05773500 | 2.32451700 | -2.95485300 |
| C | -1.05673200 | -4.87184300 | 0.86603300 |
| H | -1.65478100 | -5.53869100 | 0.22159300 |
| H | 0.01062900 | -5.09050100 | 0.69045800 |
| H | -1.28834900 | -5.10974200 | 1.91834400 |
| C | 1.19748000 | -0.59849100 | 3.23296200 |
| H | 2.15440000 | -0.81452100 | 2.72849900 |
| H | 1.29064800 | 0.38470500 | 3.72592200 |
| H | 1.02211300 | -1.36053500 | 4.01184000 |
| C | -5.86237600 | -2.75778200 | 1.35092900 |
| H | -5.71394800 | -3.22129000 | 2.34123500 |
| H | -6.74864100 | -2.10270200 | 1.40664400 |
| H | -6.07245200 | -3.55969800 | 0.62261000 |
| C | -0.65042800 | -2.61458800 | -3.92916300 |
| H | 0.36764800 | -3.03258500 | -3.84534500 |
| H | -1.34866000 | -3.44616400 | -4.12670100 |
| H | -0.67205000 | -1.93185100 | -4.79606800 |
| C | -5.11240000 | 0.25909900 | -3.12895400 |
| H | -5.54657200 | -0.63814900 | -3.60267900 |
| H | -5.93700400 | 0.84612800 | -2.68911400 |
| H | -4.63778500 | 0.86763800 | -3.91797500 |
| C | -4.65494500 | 3.47006500 | -0.38322400 |
| H | -5.57966200 | 2.89428800 | -0.20172700 |
| H | -4.65180800 | 4.33127400 | 0.30738900 |
| H | -4.68865400 | 3.86131900 | -1.41482400 |

| | | | |
|---|---|---|---|
| C | -3.74599000 | 1.20516100 | 4.17490600 |
| H | -4.78290500 | 1.57103400 | 4.08184900 |
| H | -3.73581700 | 0.38143900 | 4.90915100 |
| H | -3.12524100 | 2.02678800 | 4.57157200 |
| C | -0.07449700 | 5.56321600 | -0.69262300 |
| H | 0.94315900 | 5.95633000 | -0.51490900 |
| H | -0.26734900 | 5.60642900 | -1.77923500 |
| H | -0.79359200 | 6.23697000 | -0.19236600 |
| C | 0.05049000 | 3.71784500 | 1.81182100 |
| H | -0.65123900 | 4.38392200 | 2.34486900 |
| H | -0.09606400 | 2.69101800 | 2.18463400 |
| H | 1.07747700 | 4.03826200 | 2.06708200 |
| O | 6.81491800 | 0.35921800 | -0.75831900 |
| C | 7.14586200 | 0.02142300 | 0.36716000 |
| C | 6.68282200 | -1.25565800 | 1.09445600 |
| C | 3.42264500 | -1.80450100 | -0.62108300 |
| H | 6.31062300 | -0.96626000 | 2.09260700 |
| C | 4.43819800 | -1.14504400 | 0.33457400 |
| H | 3.21215200 | -2.81290900 | -0.20188000 |
| O | 2.22691400 | -1.08551500 | -0.62767600 |
| O | 5.60894400 | -1.85525700 | 0.33643400 |
| O | 4.24518700 | -0.15116300 | 1.01707400 |
| C | 7.79955300 | -2.29727200 | 1.19911900 |
| H | 8.16109200 | -2.58370900 | 0.19705900 |
| H | 8.64141900 | -1.88408700 | 1.77781300 |
| H | 7.42501500 | -3.19990800 | 1.70964200 |
| C | 3.99675900 | -1.97899200 | -2.04129200 |
| H | 4.27506200 | -1.00303200 | -2.47152000 |
| H | 4.89133200 | -2.62166200 | -2.02129700 |
| H | 3.22820000 | -2.44316100 | -2.68112200 |
| O | 8.00690600 | 0.70759300 | 1.16144700 |
| C | 8.52694800 | 1.92816700 | 0.57603000 |
| H | 9.07731100 | 1.70630400 | -0.35271800 |
| H | 7.70279600 | 2.62387000 | 0.35014400 |
| H | 9.19774000 | 2.35100400 | 1.33659000 |
